# Novel JAZ co‐operativity and unexpected JA dynamics underpin *Arabidopsis* defence responses to *Pseudomonas syringae* infection

**DOI:** 10.1111/nph.13683

**Published:** 2015-10-02

**Authors:** Marta de Torres Zabala, Bing Zhai, Siddharth Jayaraman, Garoufalia Eleftheriadou, Rebecca Winsbury, Ron Yang, William Truman, Saijung Tang, Nicholas Smirnoff, Murray Grant

**Affiliations:** ^1^ BiosciencesCollege of Life and Environmental SciencesUniversity of ExeterStocker RoadExeterEX4 4QDUK; ^2^ College of Biological SciencesChina Agricultural UniversityBeijing100093China; ^3^ Department of Plant BiologyUniversity of MinnesotaSaint PaulMN55108USA

**Keywords:** *Arabidopsis thaliana*, hormone, jasmonic acid (JA), JAZ, *Pseudomonas syringae*

## Abstract

- Pathogens target phytohormone signalling pathways to promote disease. Plants deploy salicylic acid (SA)‐mediated defences against biotrophs. Pathogens antagonize SA immunity by activating jasmonate signalling, for example *Pseudomonas syringae* pv. tomato DC3000 produces coronatine (COR), a jasmonic acid (JA) mimic. This study found unexpected dynamics between SA, JA and COR and co‐operation between JAZ jasmonate repressor proteins during DC3000 infection.
- We used a systems‐based approach involving targeted hormone profiling, high‐temporal‐resolution micro‐array analysis, reverse genetics and mRNA‐seq.
- Unexpectedly, foliar JA did not accumulate until late in the infection process and was higher in leaves challenged with COR‐deficient *P. syringae* or in the more resistant JA receptor mutant *coi1*. *JAZ* regulation was complex and COR alone was insufficient to sustainably induce *JAZ*s.
- JAZs contribute to early basal and subsequent secondary plant defence responses. We showed that JAZ5 and JAZ10 specifically co‐operate to restrict COR cytotoxicity and pathogen growth through a complex transcriptional reprogramming that does not involve the basic helix‐loop‐helix transcription factors *MYC2* and related *MYC3* and *MYC4* previously shown to restrict pathogen growth. mRNA‐seq predicts compromised SA signalling in a *jaz5/10* mutant and rapid suppression of JA‐related components on bacterial infection.

## Introduction

Successful infection of plants by bacterial pathogens is mediated by bacterial ‘effector’ proteins targeted to the host cell via a specialized type III secretion apparatus and by small‐molecule virulence factors. These virulence components function collectively to suppress innate immunity activated by plant pattern recognition receptor recognition of microbe‐associated molecular patterns (MAMPs; reviewed in Macho & Zipfel, 2014; Zipfel, 2014) and modify host metabolism to enhance pathogen fitness. Pathogen modulation of phytohormones by effectors has emerged as a key pathogen virulence strategy. The hormones salicylic acid (SA), jasmonic acid (JA) and ethylene (ET) have been associated with plant immunity. SA is generally considered to be important for defence against biotrophs or hemibiotrophs, such as the bacterium *Pseudomonas syringae*. JA and ET signalling are singly or collectively important for immunity to a range of necrotrophs (reviewed in Glazebrook, 2005). Recently, other hormones, such as abscisic acid (ABA), auxin and gibberellins, have been implicated in moulding defence responses (Grant & Jones, 2009; Robert‐Seilaniantz *et al*., 2011; Pieterse *et al*., 2012).

Genetic studies have revealed that jasmonates antagonize SA‐mediated biotrophic plant defences (Kloek *et al*., 2001; Brooks *et al*., 2005), although jasmonates have also been reported to synergize SA defence (Mur *et al*., 2006; Truman *et al*., 2007). The virulent hemibiotroph *P. syringae* pv. tomato strain DC3000 (DC3000) rapidly induces ABA biosynthesis in *Arabidopsis thaliana*, which also antagonizes SA accumulation (de Torres Zabala *et al*., 2007, 2009).

Some pathogens have evolved the capacity to produce hormones or hormone mimics (Grant & Jones, 2009; Robert‐Seilaniantz *et al*., 2011). The non‐host‐specific chlorosis inducing the polyketide toxin coronatine (COR) is synthesized by some (tomato, glycinea, atropurpurea, morsprunorum and maculicola) *P. syringae* pathovars (Ichihara *et al*., 1977; Bender *et al*., 1999). COR, formed by the ligation of two distinct structural compounds, coronamic acid (CMA) and coronafacic acid (CFA) (Bender *et al*., 1998), mimics the bioactive plant jasmonate (3R,7S)‐jasmonoyl‐l‐isoleucine (JA‐Ile; Katsir *et al*., 2008; Fonseca *et al*., 2009b). It promotes opening of stomata, aiding bacterial entry to the apoplast, facilitates apoplastic bacterial multiplication, contributes to disease symptoms and promotes systemic susceptibility (Brooks *et al*., 2005; Cui *et al*., 2005; Melotto *et al*., 2006).

COR has been instrumental in the genetic dissection of jasmonate signalling pathways, including the discovery of the coronatine insensitive 1 (COI1) jasmonate receptor. However, our understanding of the extent to which COR mimics jasmonates in plant defence and its *in planta* targets remains rudimentary. COR is a multifunctional defence suppressor. COR's ability to suppress host defences is partly associated with antagonism of SA signalling via COI1 activation (Kloek *et al*., 2001; Zhao *et al*., 2003); however, COR also suppresses callose deposition and promotes DC3000 multiplication in a COI1‐independent manner (Geng *et al*., 2012).

The best‐characterized JA response pathway in *A. thaliana* is mediated by the transcription factor MYC2 (Lorenzo *et al*., 2004; Chini *et al*., 2007). MYC2 is held in a transcriptionally inactive state through binding of JAZ (jasmonate–ZIM domain‐containing) repressor proteins. In the presence of JA‐Ile or COR, JAZs are ubiquitinated by the E3 ubiquitin ligase complex (SCF^COI1^) and then degraded by the 26S proteasome, freeing MYC2 to activate jasmonate signalling networks. MYC2 also activates the transcription of *JAZ*s, leading to a negative feedback loop that reimposes transcriptional repression (Lorenzo *et al*., 2004; Melotto *et al*., 2006; Chini *et al*., 2007; Thines *et al*., 2007; Fonseca *et al*., 2009a). *Arabidopsis thaliana* contains at least 12 *JAZ*s, many encoding splice variants (Chung *et al*., 2010) and most capable of homo‐ and heterodimerization (Chini *et al*., 2009; Chung & Howe, 2009). This combinatorial complexity may well provide the specificity that underpins the diversity of processes regulated by jasmonates (Pauwels & Goossens, 2011; Kazan & Manners, 2012). Although MYC2 was originally identified as the target of JAZs, the number of transcription factors interacting with JAZ proteins has expanded significantly (Wasternack, 2014). However, our understanding of how individual JAZs collectively contribute to the myriad of jasmonate‐regulated responses remains limited.

This study explores the dynamics, interaction and contribution of JA, COR and *JAZ*s to DC3000 disease progression. Inconsistent with a scenario in which JA antagonizes SA signalling, JA accumulated very late in the infected leaf, suggesting that host defence mechanisms temper JA antagonism of basal defence. *JAZ* transcripts showed a complex regulatory pattern, differentially contributing to induced basal immunity to MAMPs and a rapid and sustained induction in response to DC3000. Detailed genetic analyses revealed that JAZ5 and JAZ10 function co‐operatively to attenuate phytotoxicity mediated by COR and to moderately restrict bacterial growth. Collectively, the induction of *JAZ*s concomitant with *in planta* COR production and enhanced JA in the absence of COR suggest that plants may actively respond to bacterial COR via the sustained activation of JAZ‐based defences.

## Materials and Methods

### *Arabidopsis thaliana* growth

*Arabidopsis thaliana* (L.) Heynh. genotypes were sown in Levington F2 compost with sand and stratified for 2 d at 4°C. Plants were grown under short days at 65% humidity in a controlled environment chamber (10 h light, 120 μmol m^−2^ s^−1^, at 22°C day, 20°C night) for 5 wk before use. The Arabidopsis genotypes studied in this work were Col‐0, *sid2‐*1 (Wildermuth *et al*., 2001), *pad4‐*1 (Jirage *et al*., 1999), *myc2* and 35S::*MYC2* (Lorenzo *et al*., 2004) and a set of single, double and triple *jaz* knockout lines, the parental lines derived from the Nottingham Arabidopsis Stock Centre. Details of the lines and genotyping primers are provided in Supporting Information Table S1.

### *Pseudomonas* infections

Bacterial cultures were maintained, prepared and inoculated in Kings B medium as described previously (de Torres *et al*., 2006). For RNA and metabolite extractions and growth curves, leaves were inoculated with a 1‐ml needleless syringe on their abaxial surface with a bacterial suspension adjusted with 10 mM MgCl_2_ to a final optical density at 600 nm (OD_600_) of 0.15 or as indicated in the figure legends. All bacterial growth measurements were determined from a minimum of five independent replicates, each comprising three challenged leaves per plant. Significant growth differences between treatments were determined by Students *t*‐test (*P *<* *0.5), error bars representing the standard deviation (SD) of the mean. All experiments were repeated at least three times.

### Hormone measurements

Each hormone determination was measured in triplicate with each replicate consisting of a minimum of six infected leaves from two plants. Inoculum densities were as described in the figure legends. Samples were collected at appropriate times, frozen immediately in liquid nitrogen and subsequently freeze–dried. Hormone extractions were performed on 10 mg of powdered, freeze–dried tissue, exactly as detailed in Forcat *et al*. (2008). Figures are representative of at least two replicated experiments. Significant differences between treatments were determined by one‐way ANOVA using the least‐significant difference *post‐hoc* test, error bars representing the SD of the mean. Methyl jasmonate (MeJA) and JA‐l‐Ile were purchased from OLCHEMIM (Olomouc, Czech Republic). CFA was a gift from Robin Mitchel (HortResearch Auckland, New Zealand).

### RNA extraction and quantitative PCR analysis

Total RNA extraction and quantitative reverse transcription‐polymerase chain reaction (qRT‐PCR) were performed as described by de Torres Zabala *et al*. (2009). *Actin 2* (At3g18780) was used as internal standard to normalize cDNA abundance between samples. Relative expression levels are expressed in arbitrary units using non‐inoculated Col‐0 plants as being equivalent to unity. The primers used for qRT‐PCR and the size of the resulting amplicons are provided in Methods S1. For *JAZ10* splice variants, a common forward primer (*JAZ10*: F 5′‐AGCCTCAGATCCCGATTTCTC‐3′) and specific reverse primers (*JAZ10.1*: R 5′‐GATGTTGATACTAATCTCTCCTTG‐3′; amplicon size, 334 bp; *JAZ10.3*: R 5′‐GGATTGTTGAAGAATCATTACCTC‐3′; amplicon size, 341 bp; *JAZ10.4*: R 5′‐CGATGGGAAGATCGAAAGATC‐3′; amplicon size, 218 bp) were used.

For the measurement of *P. syringae* gene expression, *GYR‐A* was used as a reference housekeeping gene. Graphs show the means and error bars (± SD) of three biological replicates. All RT‐PCR experiments were performed at least twice with similar results.

### CATMA microarrays

For RNA expression studies, bacterial inoculum densities were adjusted to OD_600_ = 0.15. For each treatment, mock, DC3000 or DC3000*hrpA*, four biological replicates comprising leaf 8 of 4‐wk‐old plants were syringe infiltrated. Samples were collected at 0, 2, 3, 4, 6, 7, 8, 10, 12, 14 and 17.5 h post‐inoculation (hpi) and snap frozen in liquid nitrogen. RNA was extracted and hybridized to CATMA arrays (Allemeersch *et al*., 2005) and processed as described previously (Breeze *et al*., 2011). The data comprise the means from four single‐leaf biological replicates and two technical replicates per time point. Blue and yellow matrices indicate that differential expression is significantly (Student's *t*‐test *P = *0.01, *n *=* *8) induced or suppressed, respectively, between treatments. The full infection time course microarray analysis data are deposited at Gene Expression Omnibus (GEO) under the accession number GSE56094.

### Root assays

Surface‐sterilized wild‐type and various combinations of *jaz* mutants were germinated on 25‐cm^2^ plates containing half‐strength Murashige and Skoog (MS) medium supplemented with either COR (Sigma) at 0.02 or 0.2 μM, or MeJA (Sigma) at 10 or 50 μM. Plates were incubated in an upright position under short days (see the ‘*Arabidopsis thaliana* growth’ subsection above) and the root length of 8‐d‐old seedlings was measured using ImageJ software (http://imagej.nih.gov/ij/).

### mRNA‐seq

Total RNA from naïve or DC3000‐challenged leaves (OD_600_ = 0.15) of Col‐0 or *jaz5/10* plants was isolated at 6, 8, 12 or 16 hpi. Three leaves from four plants were collected at each time point and RNA was prepared as described earlier. mRNA‐seq libraries were prepared using Poly(A)‐RNA, pooled from three biological replicates for each time point. Directional RNA libraries were prepared using Illumina's (San Diego, CA, USA) ScriptSeq v.2 protocol, and library size and concentrations were calculated using a Bioanalyser DNA7500 chip. Libraries were pooled in equimolar amounts, denatured and diluted to 6.5 pM, clustered and 100‐bp paired‐end sequenced on an Illumina HiSeq 2500 using Illumina SBS reagents. Data were analysed by gFOLD (Feng *et al*., 2012) using a cutoff of log_2_ 1.6. Data are available from GEO under the submission number GSE72461.

## Results

### Hormone and COR accumulation during DC3000 infection

**Figure 1:**
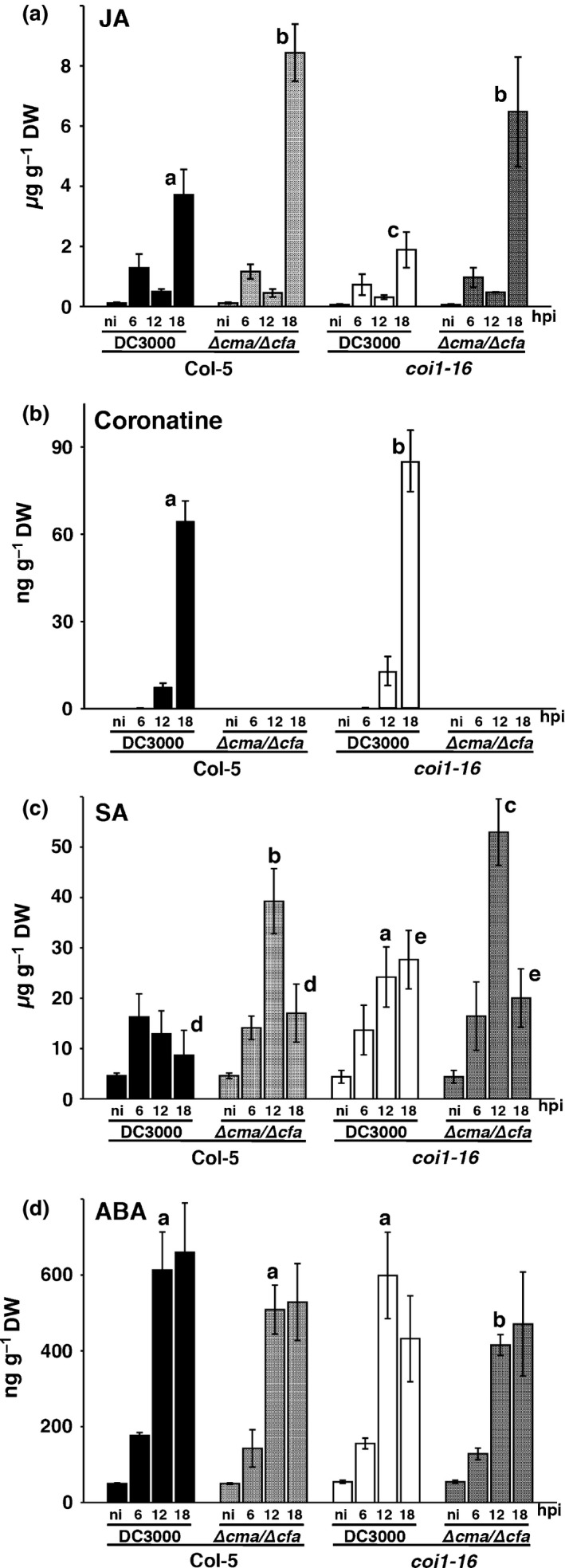
Impact of coronatine and the coronatine insensitive 1 (COI1) JA receptor on phytohormone dynamics during *Pseudomonas syringae* DC3000 disease progression in *Arabidopsis thaliana*. Wild‐type Col‐5 and the *coi1*‐16 jasmonate receptor mutant were syringe inoculated with virulent DC3000 or the coronatine‐deficient DC3000∆*cfa6*:∆*cma* (∆*cfa6/*∆*cma*) strain (OD_600_ = 0.15) and foliar levels of (a) jasmonic acid (JA), (b) coronatine, (c) salicylic acid (SA) and (d) abscisic acid (ABA) were determined at 6, 12 and 18 h post‐inoculation (hpi), as well as non‐inoculated tissue (ni). Different letters indicate significant differences (*P *<* *0.05) between the corresponding treatments at that time point as assessed by one‐way ANOVA using the least‐significant difference (LSD) *post‐hoc* test (means ± SD;* n *=* *3). Experiments were repeated at least three times and all showed the same trend.

We measured JA, ABA, SA and COR in wild‐type *A. thaliana* (Col‐5) and the JA receptor mutant *coi1‐*16 (cleared of the *pen2* mutation; Westphal *et al*., 2008), which is compromised in most jasmonate responses (Turner *et al*., 2002), following infection with either *P. syringae* DC3000 or strain DC3000∆*cfa6*:∆*cmaA*, which is deficient in the production of both COR precursors, CFA and CMA (Brooks *et al*., 2004). JA rather than JA‐IIe was measured because DC3000 responses are wild‐type in *jar1* mutants (Staswick & Tiryaki, 2004; Laurie‐Berry *et al*., 2006), although JA acts as a proxy for many active jasmonates and JA‐Ile dynamics often mirror JA dynamics (Balcke *et al*., 2012). The DC3000*hrpA* mutant, which activates plant basal defence, but is unable to deliver effectors or to synthesize appreciable quantities of COR (de Torres Zabala *et al*., 2009), did not induce appreciable JA accumulation (Fig. S1a) compared with virulent DC3000 (Fig. 1a). Indeed, JA did not accumulate until 18 hpi in all challenges (Fig. 1a), whereas COR first accumulated between 6 and 10 hpi in DC3000‐inoculated leaves (Fig. 1b). Unexpectedly, at 18 hpi, JA accumulated to twice the level in leaves challenged with DC3000∆*cfa6*:∆*cmaA* compared with DC3000 (Fig. 1a). Rather than stimulating (Cui *et al*., 2005; Laurie‐Berry *et al*., 2006), COR appears to directly or indirectly compromise JA accumulation (Fig. 1a).

In *coi1‐*16, JA levels were *c*. 50% of wild‐type levels (Fig. 1a), indicating that COI1‐independent processes contribute to JA accumulation. By contrast, JA levels in DC3000∆*cfa6*:∆*cmaA*‐challenged *coi1‐*16 leaves were similar to those in DC3000∆*cfa6*:∆*cmaA*‐challenged Col‐5. Together, these results suggest that JA accumulation following DC3000∆*cfa6*:∆*cmaA* challenge is largely COI1 independent. JA levels in the 2‐oxophytodienoic acid reductase *opr3* JA biosynthetic mutant (Stintzi & Browse, 2000) were *c*. 80‐fold lower than in the parental Ws‐0 ecotype. Thus, DC3000‐induced JA is most probably derived from *de novo* synthesis (Fig. S2).

SA levels were maximal at 6 hpi in DC3000‐challenged Col‐5 and decreased thereafter, consistent with effector‐mediated suppression of SA (Fig. 1c). At 12 hpi, SA levels were significantly higher in Col‐5 leaves challenged with DC3000∆*cfa6*:∆*cmaA* (*c*. 2.5‐fold) or *coi1*‐16*/*DC3000∆*cfa6*:∆*cmaA* (*c*. three‐fold) compared with Col‐5/DC3000 inoculation. The marked decrease in SA levels between 12 and 18 h in leaves challenged with DC3000∆*cfa6*:∆*cmaA* (Fig. 1c) parallels the concomitant increase in JA seen in both genotypes at this time (Fig. 1a). However, in DC3000/*coi1‐*16‐infected leaves, SA levels only differed significantly from DC3000/Col‐5 challenge at 18 hpi. These data suggest that COR‐dependent suppression of SA during DC3000 infection is not fully COI1 dependent. Neither COR nor COI1 markedly affected the rapid (6 hpi) ABA accumulation (de Torres Zabala *et al*., 2009), which reached maximal levels at 12 hpi (Fig. 1d). ABA levels only differed from the wild‐type in *coi1*‐16*/*DC3000∆*cfa6*:∆*cmaA*, where they were slightly lower.

In summary, foliar JA accumulates unexpectedly late in the infection process, well after bacterial multiplication occurs. A significant proportion of this JA accumulation is COI1 independent and, counter‐intuitively, antagonized by COR production. This pattern of antagonism is consistent with COR triggering a host defence response.

### Induction of transcripts encoding JA biosynthetic components during a compatible interaction

**Figure 2:**
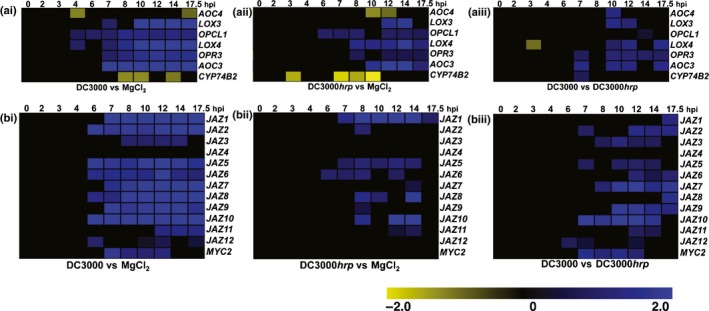
Expression dynamics of *Arabidopsis thaliana* jasmonic acid (JA) biosynthetic and *JAZ* genes during responses to virulent *Pseudomonas syringae* DC3000 or the non‐pathogenic DC3000*hrpA* mutant. Differential expression of (a) jasmonate biosynthetic genes and (b) the 12 *JAZ* genes and *MYC2* over a 17.5‐h time course, reporting the response to (i) virulent DC3000, (ii) a disarmed DC3000*hrp* mutant or (iii) the activity of virulence factors (DC3000 vs DC3000*hrp*). Gene expression levels from samples collected at the stated hours post‐inoculation (hpi) were determined using CATMA microarrays (Allemeersch *et al*., 2005) and data extraction and normalization as described previously (Breeze *et al*., 2011). Data represent the means of four biological replicates of leaf 8 and two technical replicates per time point (see Supporting Information Table S1). Bacterial inoculum was OD_600_ = 0.15. Blue and yellow matrices indicate that differential expression is significantly induced or suppressed, respectively, between treatments (pairwise *t*‐test, *P < *0.01, *n *=* *8), with black representing no significant change.

We next examined the expression of genes related to jasmonate signalling in a high‐resolution microarray experiment, reporting *A. thaliana* responses to virulent DC3000, DC3000*hrpA* and mock (MgCl_2_) challenges (Fig. 2a; Table S2). We compared JA biosynthetic genes induced during basal defence (DC3000*hrpA* vs MgCl_2_; Fig. 2ai) with a compatible interaction (DC3000 vs MgCl_2_; Fig. 2aii), using mock challenge to remove any wound response induced by syringe infiltration (de Torres Zabala *et al*., 2009; Fig. S1). JA biosynthesis pathway genes were induced within 7 hpi by DC3000 and were also significantly, but more weakly, induced by DC3000*hrpA* challenge, which does not lead to the accumulation of appreciable amounts of JA (Figs 2aiii, S1). Concomitant suppression of *CYP74B2* (At4g15440), encoding hydroperoxide lyase 1 involved in the production of hexanal and 12‐oxo‐*cis*‐9‐dodecenoic acid from 13‐hydroperoxide (Duan *et al*., 2005), potentially increases substrate availability for JA synthesis following DC3000 challenge.

### Induction of JAZ transcriptional repressors during bacterial challenge

The disparity between early (7 hpi) transcriptional activation of JA biosynthetic genes and late (18 hpi) accumulation of *de novo* JA is striking. This result implies that active host defences alleviate JA antagonism of SA signalling and desensitize the host to COR. One possibility is that specific sustained induction of *JAZ*s may lock JAZ targets into a constitutively repressed state. The analysis of *JAZ* and *MYC2* expression profiles (Fig. 2b; Table S2) revealed that *MYC2* and most *JAZ*s, with the exception of *JAZ*s *4*,* 11* and *12*, were rapidly induced by DC3000 between 6 and 7 hpi (Fig. 2bi), albeit with contrasting dynamics. Generally *JAZ*s (and *MYC2*) were more strongly induced by DC3000. *JAZ1*,* JAZ5* and *JAZ6* (but not *MYC2*) most rapidly responded to DC3000*hrpA* challenge (Fig. 2bii), whereas *JAZ*s *1* and *8* had a strong DC3000*hrpA* profile and were only significantly different from DC3000 at 17.5 hpi (Fig. 2biii).

### JAZ5 and JAZ10 collaborate to restrict COR phytotoxicity

**Figure 3:**
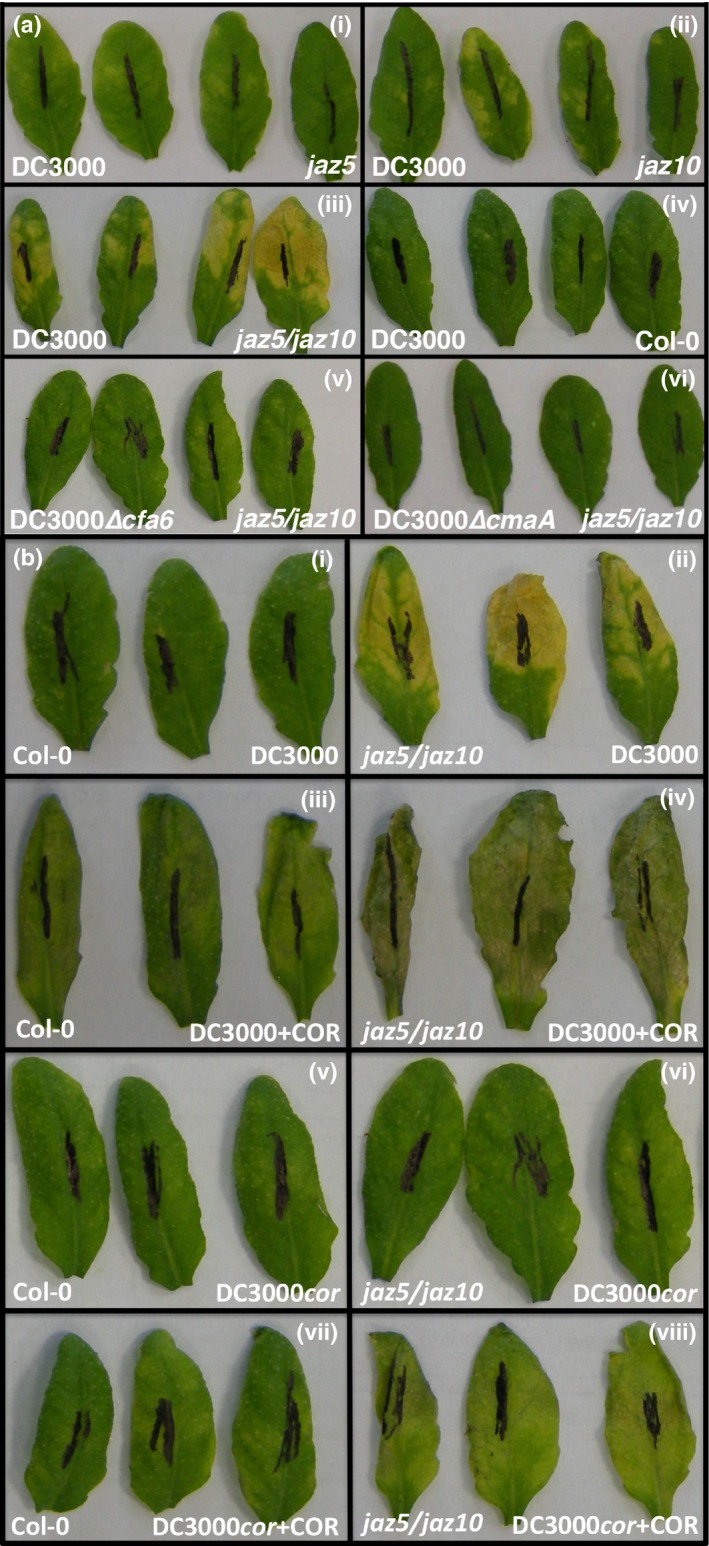
*Arabidopsis thaliana* JAZ5 and JAZ10 collaborate to restrict coronatine (COR)‐mediated virulence. A *jaz5/10* double mutant, but neither single mutant alone, exacerbates the phytotoxic effects of COR. (a) Infection phenotypes at 5 d post‐inoculation (dpi) in (i) *jaz5*, (ii) *jaz10* and (iii) *jaz5/10* leaves following challenge with *Pseudomonas syringae* DC3000 (OD_600_ = 0.0005) compared with wild‐type Col‐0 (iv). Occasional enhanced chlorosis in *jaz10*‐challenged leaves is illustrated for completeness. No chlorosis was evident in *jaz5/10* infected with either the *Δcfa6* or *ΔcmaA* DC3000 mutants (v, vi). Experiments were repeated more than six times with identical results. (b) *jaz5/10* plants are more sensitive to bacterial COR. Infection phenotypes of wild‐type or the *jaz5/10* mutant following co‐infiltration with DC3000 (i, ii) or DC3000 plus COR (iii, iv). Infiltration of COR induced strong necrotic symptoms in *jaz5/10* leaves indicative of hypersensitivity to COR (iv). In wild‐type Col‐0, co‐infiltration of COR with DC3000∆*cfa6*:∆*cmaA* (DC3000*cor*) did not lead to more symptoms (vii) compared with DC3000*cor* challenge alone (v). By contrast, compared with DC3000*cor* challenge (vi), leaves of the *jaz5/10* mutant co‐infiltrated with COR and DC3000*cor* showed significant hypersensitivity (viii). Bacterial inoculations were at a density of OD_600_ = 0.0005 and photographs were taken at 5 dpi. For co‐infiltration challenges in (b), COR (0.25 μM) was infiltrated first with the bacterial suspension and then again 2 d later. This experiment was repeated four times with similar results.

This almost synchronous induction of *JAZ*s coincided with the first detectable levels of COR (Fig. 1b; de Torres Zabala *et al*., 2009). We used a reverse genetic approach to investigate the possible role of *JAZ*s in the enhanced JA accumulation in the absence of COR production (Fig. 1a,b). Arabidopsis T‐DNA insertion mutants in *JAZ*s *1–7, JAZ10* and *JAZ12* showed wild‐type symptoms following DC3000 challenge, with the exception of mildly enhanced chlorosis in *jaz10* leaves (Fig. 3aii), as reported previously (Demianski *et al*., 2012). To address possible functional co‐operation between JAZs, we generated a collection of double mutants (Table S1) and screened for altered symptoms. Strikingly, the *jaz5/10* double mutant (see Fig. S3 for transcript accumulation in mutants) showed strong chlorosis at 5 d post‐inoculation (dpi) following either syringe infiltration with DC3000 (Fig. 3aiii) or dip inoculation, which was not evident in either single mutant (Fig. 3ai,aii) or in any other double mutant combination tested (Table S1). The incorporation of additional knockouts of *JAZ2, 3*,* 6* or *7* into the *jaz5/10* background did not further enhance leaf chlorosis (Table S1). Remarkably, chlorotic symptoms were completely abolished in *jaz5/10* plants challenged with DC3000*cor* mutants (Fig. 3av,avi). The COR toxin induces chlorosis and *jaz5/10* leaves co‐inoculated with COR (0.25 μM) and DC3000 (Fig. 3b) showed stronger symptom development compared with Col‐0 (Fig. 3biii,biv), indicating enhanced COR sensitivity. Co‐infiltration of DC3000∆*cfa6:∆cmaA* with COR (0.25 μM) partly restored the chlorosis phenotype in *jaz5/10*, but not Col‐0 (Fig. 3bvii,bviii), whereas DC3000∆*cfa6:∆cmaA* challenge alone failed to cause symptoms in either genotype (Fig. 3bv,bvi). Collectively, these results demonstrate a co‐operative role for JAZ5 and JAZ10 in suppressing the phytotoxic effects of COR.

**Figure 4:**
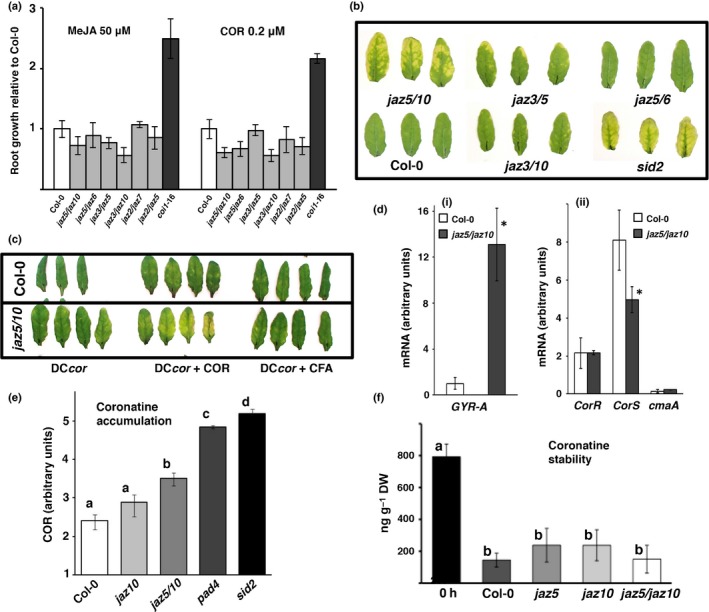
The relationship between chlorosis, root growth, coronatine (COR) production, COR stability and COR regulation in *Arabidopsis thaliana* wild‐type and *jaz5/10* mutants. (a) Jasmonate sensitivity of *jaz5/10*. Seedling root growth of various double *jaz* mutant combinations (see Table S1) and *coi1‐*16 on half‐strength Murashige and Skoog (MS) medium supplemented with methyl jasmonate (MeJA) (50 μM) or COR (0.2 μM), determined relative to Col‐0. Roots were measured 8 d after sowing using ImageJ. (b) Leaf infection phenotypes of *jaz3/5, jaz5/6* and *jaz3/10* compared with *jaz5/10* and a *sid2* control following *Pseudomonas syringae* DC3000 challenge (OD_600_ = 0.002). Leaves were photographed at 4 d post‐inoculation (dpi). (c) Chlorosis is caused by bioactive COR. Co‐infiltration of leaves of *jaz5/10* with DC3000*∆cfa6/cmaA* (DC *cor*) and COR (0.25 μM), but not CFA (0.25 μM), the polyketide and biosynthetic intermediate of COR, resulted in enhanced chlorosis (4 dpi). (d) COR regulation/synthesis is not modified in *jaz5/jaz10* mutants. Total RNA was extracted from leaves challenged with DC3000 (OD_600_ = 0.002) at 3 dpi and steady‐state mRNA levels were quantified by reverse transcription‐polymerase chain reaction (RT‐PCR) (*n *=* *3). (di) *GYR‐A* transcript abundance reflects enhanced bacterial growth in *jaz5/10* leaves. (dii) Steady‐state mRNA of *CorR*,* CorS* (COR regulatory genes) and *cmaA* (a COR biosynthetic gene) determined using *GYR‐A* as a reference gene (means ± SD;* n *=* *3). Asterisks show significant differences in transcript abundance (*t*‐test, *P *<* *0.05) between genotypes. (e) *jaz5/10* plants do not overexpress COR. COR was measured in wild‐type Col‐0, *jaz10*,* jaz5/10*,* pad4* and *sid2* mutants at 16 h post‐inoculation (hpi) with DC3000 (OD_600_ = 0.15; means ± SD;* n *=* *3). Different letters denote significant differences in COR (*t*‐test, *P *<* *0.05) between genotypes. COR levels were significantly higher in *jaz5/10* compared with Col‐0, but substantially lower than in the hypersusceptible *pad4* and *sid2* mutants. (f) Stability of COR in *jaz5/10* lines is not altered relative to wild‐type Col‐0 or cognate *jaz5* and *jaz10* parental lines. Leaf COR was measured at 16 hpi after infiltration with 1.6 μM (500 ng ml^−1^) COR.

We compared jasmonate sensitivity of *jaz5*/*10* to various other double mutant combinations using root growth assays on medium supplemented with MeJA (5.0 μM) or COR (0.2 μM). *jaz5/10* showed no significant difference in root growth compared with most other double mutant combinations (Figs 4a, S4), yet only *jaz5/10* developed the strong chlorotic symptoms following DC3000 challenge (Fig. 4b). Chlorosis was dependent on bioactive COR, as co‐infiltration with DC3000∆*cfa6:∆cmaA* and CFA did not induce enhanced chlorosis (Fig. 4c). These results show that the co‐operative activities of JAZ5 and JAZ10 abrogate COR phytotoxicity in DC3000‐infected leaves. We next tested whether JAZ5/10 reduced sensitivity to COR by enhancing COR degradation or preventing the synthesis/transport of COR by the bacteria. We discounted the possibility that *jaz5*/10 mutants influence COR production, as levels of expression of the bacterial *Cfa6* polyketide synthase or the *CorR* regulator (Fig. 4d) were similar. Indeed, *CorS* levels were actually lower in *jaz5*/*10*. We excluded hyperaccumulation of COR, as COR was lower in *jaz5*/*10* mutants than in enhanced susceptibility *pad4* or *sid2* mutants at 16 hpi with DC3000 (Fig. 4e). Finally, exogenous application of COR did not enhance COR stability in *jaz5/10* (Fig. 4f). Thus, JAZ5 and JAZ10 can function *in planta* to restrict COR cytotoxicity.

### *jaz5/10* is moderately more susceptible and shows altered hormone levels

**Figure 5:**
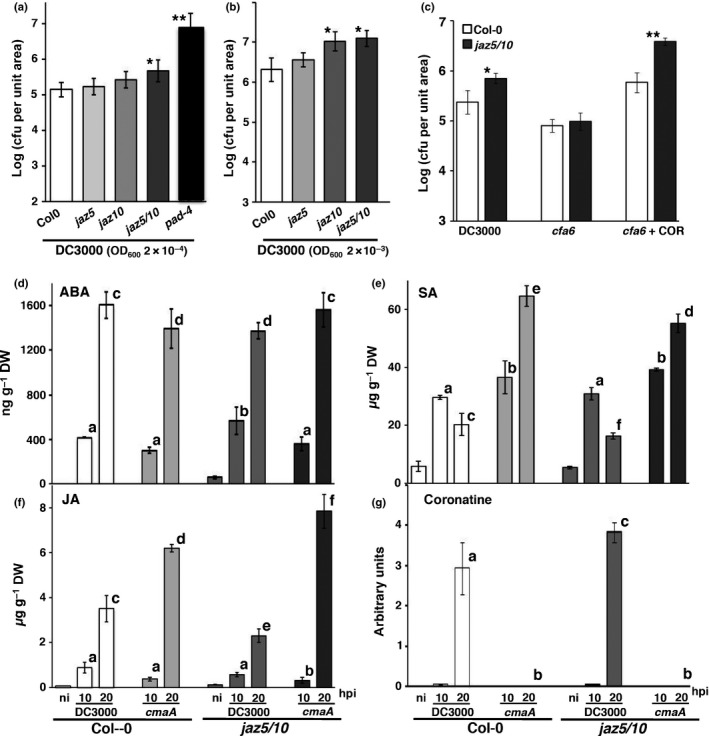
*Arabidopsis thaliana jaz5/10* mutants support greater growth of virulent *Pseudomonas syringae* DC3000 and show altered hormone profiles. (a) At low inoculum (OD_600_ = 0.0002), *jaz5/10,* but not *jaz5* or *jaz10*, plants support greater bacterial growth of virulent DC3000 compared with Col‐0 at 3 d post‐inoculation (dpi). This enhanced susceptibility is moderate compared with the hypersusceptible *pad4* mutant. (b) At high inoculum (OD_600_ = 0.002), both *jaz10* and *jaz5/10* plants support greater DC3000 growth at 3 dpi. (c) DC3000*∆cfa6* co‐infiltrated with coronatine (COR, 250 μM), but not DC3000*∆cfa6* alone, leads to hypersusceptibility in *jaz5/10* leaves at 3 dpi. Growth is represented by means ± SD (*n *=* *6, *t*‐test; *, *P *<* *0.05; **, *P *<* *0.01). (d–g) Hormone and COR levels in Col‐0 and *jaz5/10* leaves. (d) Abscisic acid (ABA), (e) salicylic acid (SA), (f) jasmonic acid (JA) and (g) COR levels were measured at 10 and 20 h post‐inoculation (hpi) after challenge with DC3000 or DC3000*cmaA* (OD_600_ = 0.15). Different letters represent significantly different treatments (*P *<* *0.05; one‐way ANOVA using the least‐significant difference (LSD) *post‐hoc* test) from their respective DC3000 Col‐0 challenge at the indicated time point (*n *=* *3; means ± SD). Non‐inoculated tissue (ni) was used to report basal hormone levels. cfu, colony‐forming unit.

*jaz5/10* supported significantly enhanced growth of DC3000 at 3 dpi at low inoculum (OD_600_ = 0.0002) compared with Col‐0, *jaz5* and *jaz10*, corroborating the observation of moderately enhanced symptoms, but not enhanced bacterial growth, in *jaz10* (Demianski *et al*., 2012). However, the increased susceptibility of *jaz5*/*10* was modest compared with the highly susceptible *pad4* mutant (Jirage *et al*., 1999) (Fig. 5a). This result suggests that JAZ5 and JAZ10 make a small, but significant, contribution to host defence against DC3000. Interestingly, at higher inoculum (OD_600_ = 0.002), both *jaz5*/*10* and *jaz10* supported more bacterial growth (Fig. 5b), suggesting that JAZ10 may restrict bacterial growth at high titre, such as experienced later in disease development. Furthermore, *jaz5/10* was more susceptible than Col‐0 to co‐infiltration of DC3000*∆cfa* with COR (Fig. 5c), indicating that JAZ5/10 moderates COR virulence.

Hormone profiles revealed complex responses. Compared with Col‐0, ABA was higher at 10 hpi and SA was lower at 20 hpi in DC3000‐challenged *jaz5*/*10*, possibly explaining its moderately increased susceptibility (Fig. 5a). As in Fig. 1, JA levels were significantly higher in DC3000*∆cmaA‐* than in DC3000‐challenged Col‐0 leaves at 20 hpi (Fig. 5f). Notably, JA was even higher in COR‐deficient DC3000*∆cmaA‐*challenged *jaz5/10* leaves compared with Col‐0 (Fig. 5g). This result indicates that JAZ5 and JAZ10 help to restrict late JA accumulation. Although COR levels were correspondingly higher in *jaz5*/*10* leaves challenged with DC3000 compared with Col‐0 (Fig. 5g), JA was moderately, but significantly, lower in DC3000‐challenged *jaz5/10* leaves. Collectively, these results reveal a complex interaction in which JAZ5/10 contributes not only to reduced cytotoxicity, but to enhanced foliar JA in the absence of COR, but restricts JA accumulation in the presence of COR.

### Bacterial COR alone is insufficient for sustained induction of *JAZ*s

**Figure 6:**
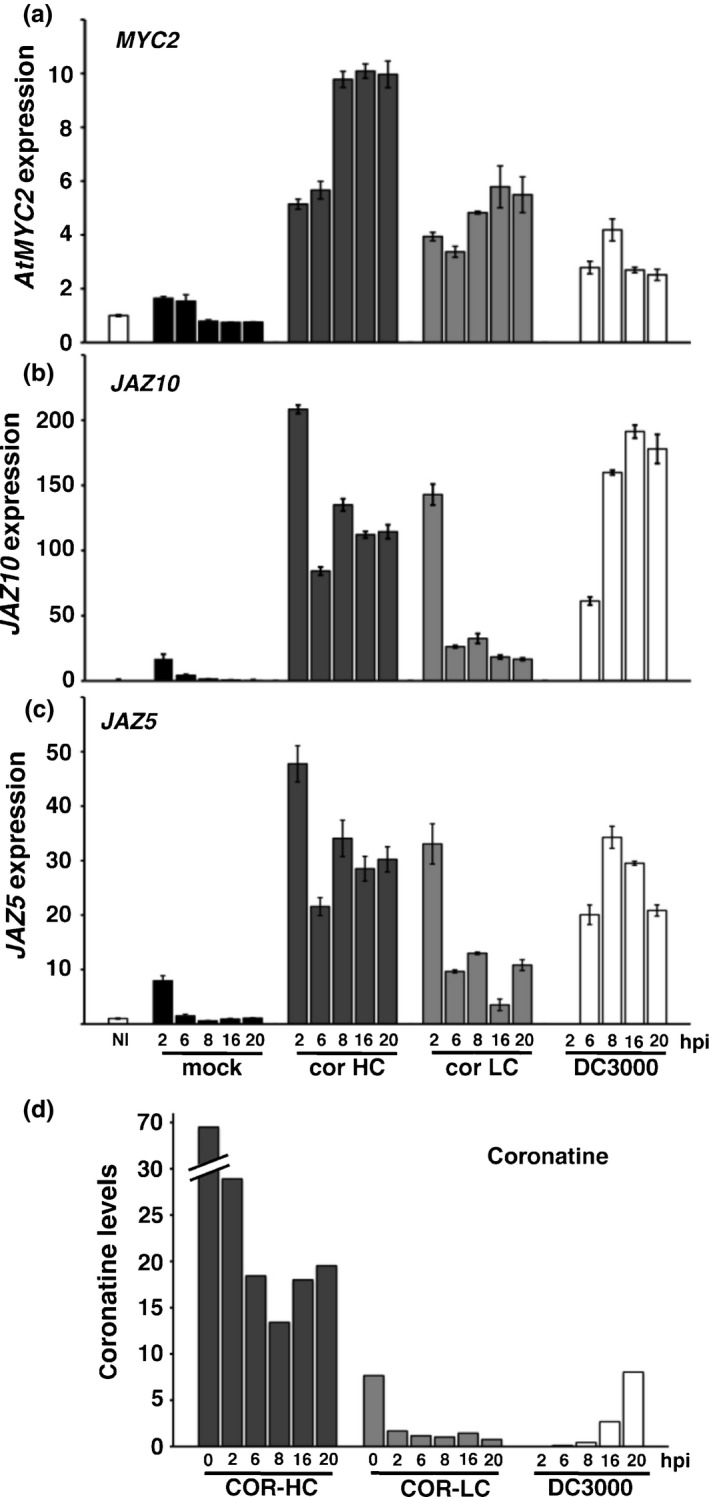
Steady‐state transcript dynamics of *Arabidopsis thaliana MYC2*,*JAZ5* and *JAZ10* in leaves challenged with *Pseudomonas syringae* DC3000 or coronatine (COR). Leaves were infiltrated with either high (16 μM; 5 μg ml^−1^) or low (1.6 μM; 500 ng ml^−1^) concentrations of COR or challenged with DC3000 (OD_600_ = 0.15). (a) COR infiltration induces a strong, sustained induction of *MYC2*, whereas DC3000 results in a weak, transient induction in challenged leaves. (b, c) COR alone cannot replicate the *JAZ10* or *JAZ5* dynamics observed during DC3000 infection. Strong sustained *JAZ* expression is evident within 6 h post‐inoculation (hpi) of DC3000 challenge, whereas COR infiltration results in a rapid, but transient, increase in *JAZ5* and *JAZ10*. For (a–c), *n *=* *3 and error bars represent ± SD. (d) Persistence of COR. COR levels determined in leaves following DC3000 challenge or infiltration with different doses of COR. Steady‐state *JAZ* transcript levels at 6 and 8 hpi were significantly higher in DC3000 compared with COR‐challenged leaves (b, c) despite less COR being present in the DC3000 challenged tissue (d). Experiments were repeated twice with similar results.

The strong chlorosis induced by DC3000 on *jaz5*/10 leaves is reminiscent of symptoms on DC3000‐infected tomato (Ishiga *et al*., 2009). The sustained *JAZ* induction following DC3000 challenge is coincident with COR production. To investigate whether COR alone activates *JAZ* transcription, we measured the expression of *JAZ5*,* JAZ10* and *MYC2* in Col‐0 following mock, DC3000 or pure COR challenge. We tested high (5 μg ml^−1^; 16 μM) and low (0.5 μg ml^−1^; 1.6 μM) COR concentrations. Low represents the maximal levels experimentally detected in DC3000‐challenged Col‐0 leaves (de Torres Zabala *et al*., 2009) (Fig. 6).

*MYC2* was not transcriptionally responsive to DC3000, only transiently increasing at 6 hpi, despite significant COR accumulation in infected leaves at 16 hpi (Fig. 6d). By contrast, *MYC2* showed sustained dynamics at both COR concentrations, notably being highest at 16–20 hpi with [COR]_low_ – despite COR being only a fraction of that found in DC3000‐challenged leaves at that time point (compare Fig. 6a and d).

*JAZ5* and *JAZ10* transcript dynamics differed between COR and DC3000 challenges (Fig. 6b–d). As in Fig. 2, *JAZs* were strongly induced at 6 hpi by DC3000, despite COR being virtually undetectable. *JAZ10* accumulation was maximal at 16 hpi (Fig. 6b), whereas *JAZ5* was maximal at 8 hpi (Fig. 6c). In contrast with the sustained *MYC2* induction in response to COR application (Fig. 6a), *JAZ5* and *JAZ10* were induced transiently, peaking at 2 hpi before rapidly declining. Despite COR levels being four‐fold lower, *JAZ5* and *JAZ10* transcript accumulation at 8 hpi with DC3000 was similar to [COR]_low_ at 2 hpi (Fig. 6d). Thus, although *JAZ5*/*10* expression was transiently induced in response to initial COR application, additional co‐stimulatory factors, such as MAMPs (Fig. 2b), appear to be necessary to sustain or synergistically enhance *JAZ5/10* expression.

### The *jaz5/10* chlorosis phenotype is not dependent on MYC2 and cannot be replicated by exogenous application of JA‐Ile or by HopX

Both JAZ5 and JAZ10 interact with MYC2 in yeast two‐hybrid assay. If the release of MYC2 induces the *jaz5/10* chlorotic infection phenotype, overexpression of *MYC2* should reproduce this phenotype. DC3000‐challenged 35S::*MYC2* overexpression lines (Lorenzo *et al*., 2004) were no more chlorotic than wild‐type plants (Fig. S5a), yet *jaz5/10* and 35S::*MYC2* supported moderately increased growth of DC3000 (Fig. S5b). Furthermore, no discernible difference in chlorosis between DC3000‐challenged *jaz5/10* and *myc2/jaz5/jaz10* (Fig. S5c) indicated that the loss of *myc2* does not attenuate the chlorotic phenotype. Taken together, these data indicate that the *jaz5*/*10* response to DC3000 expressing COR is elaborated independently of *MYC2* function. Moreover, only DC3000*cor* co‐infiltration with COR (0.2 μM), but not MeJA (50 mM) or JA‐L‐Ile (50 mM), induced strong chlorotic symptoms (Fig. S5d).

*Pseudomonas syringae* pv. tabaci 11528 does not produce COR, but encodes the cysteine protease HopX1 which interacts with and promotes the degradation of JAZ proteins in a COI1‐independent manner (Gimenez‐Ibanez *et al*., 2014). Although DEX‐inducible *HopX1* induces chlorotic symptoms and strongly upregulates *JAZ5*,* JAZ10* and *JAZ12* (Gimenez‐Ibanez *et al*., 2014), delivery of HopX1 by DC3000*cor* did not induce chlorosis in *jaz5/10* leaves (Fig. S5e).

### *JAZ5* and *JAZ10* are regulated in a complex manner that is partly dependent on COR and requires additional bacterial signals

**Figure 7:**
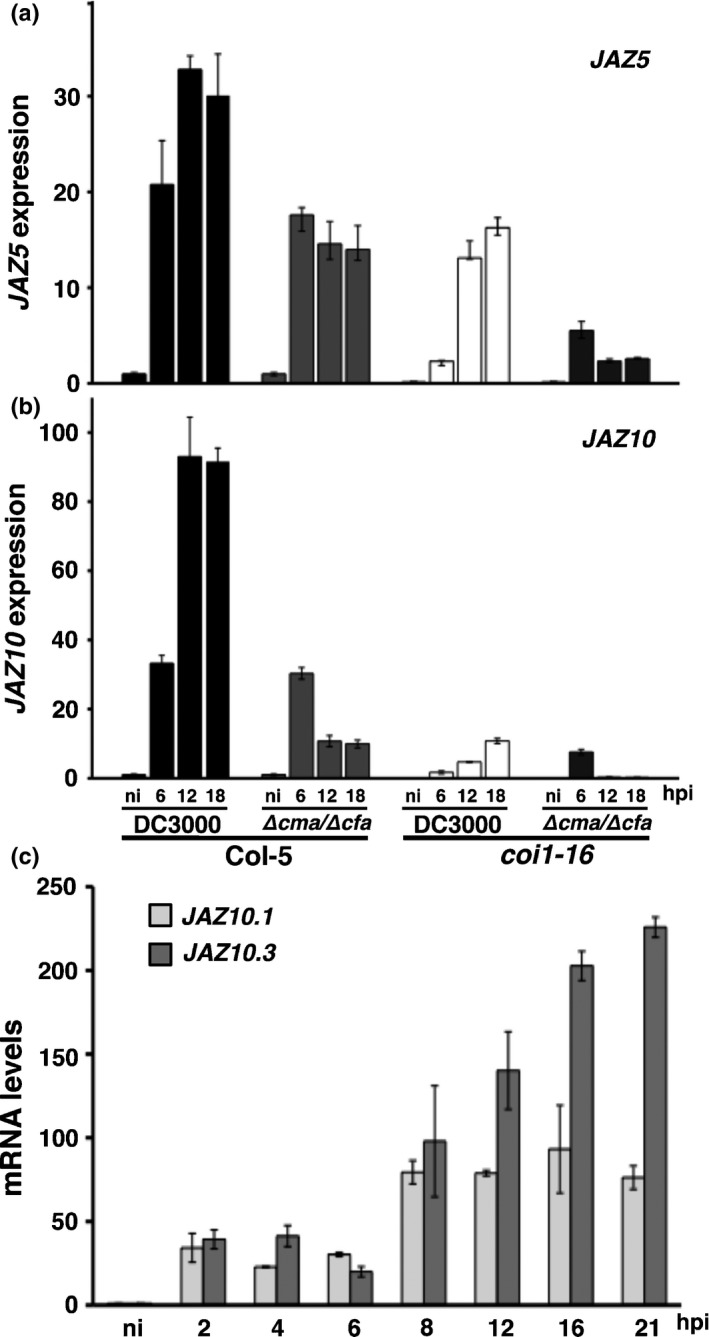
Impact of coronatine and the coronatine insensitive 1 (COI1) jasmonic acid (JA) receptor on the temporal dynamics of *Arabidopsis thaliana JAZ5* and *JAZ10* expression following *Pseudomonas syringae* DC3000 challenge. Temporal expression profiles of (a) *JAZ5* and (b) *JAZ10* were determined in Col‐0 wild‐type and the JA receptor mutant *coi1*‐16 following challenge with DC3000 or the coronatine‐deficient DC3000∆*cfa6*:∆*cma* strain. (c) Differential accumulation of *jaz10* splice variants during the infection process. Transcripts encoding *jaz10.1* and *jaz10.3* were measured by quantitative reverse transcription‐polymerase chain reaction (qRT‐PCR) during infection of Col‐0 leaves with DC3000 (*n *=* *3, means ± SD). ni, non‐induced; hpi, hours post‐inoculation. qRT‐PCR experiments were repeated twice with similar results.

We next compared *JAZ5* and *JAZ10* dynamics during infection by qRT‐PCR. Initial *JAZ5* and *JAZ10* transcript accumulation at 6 hpi was similar in DC3000 and DC3000∆*cfa6*:∆*cmaA*, indicative of a COR‐independent MAMP response (Fig. 7a,b). Subsequent COR accumulation contributed to *c*. 50% of *JAZ5* and *c*. 80% of *JAZ10* induction at 12 and 18 hpi, respectively (Fig. 7a,b; compare DC3000 vs DC3000∆*cfa6*:∆*cmaA*). Interestingly, COI1 was required for nearly all *JAZ10,* but only *c*. 50% of *JAZ5*, transcript accumulation (Fig. 7b), suggesting that COR induction of *JAZ10* is primarily achieved through COI1.

Both JAZ5 and JAZ10 are required to protect against COR phytotoxicity. Congruently, *JAZ5* and, particularly, *JAZ10* expression is dependent on both COR and additional stimuli, most probably bacterial‐derived signals, as COR alone cannot sustain the expression of these two *JAZ*s (Fig. 6). Differential splicing of *JAZ10* can lead to splice variants lacking the Jas domain PY motif, resulting in enhanced resistance to ubiquitin‐mediated proteasomal degradation (Chung *et al*., 2010). Following DC3000 challenge, we detected *JAZ10.1* and *JAZ10.3*, but not *JAZ10.4* (which accumulates during the wound response; Chung & Howe, 2009). The sustained *JAZ10* increase during infection (Fig. 6b) is largely attributable to *JAZ10.3* from 8 hpi (Fig. 7c). JAZ10.3 is predicted to bind to COI1, but is not efficiently ubiquitinated, therefore attenuating JA signalling (Chung *et al*., 2010). Both sustained increases in *JAZ*s and specific accumulation of the ubiquitination‐resistant JAZ10.3 isoform are complementary mechanisms capable of modulating JA signalling.

### Transcriptional changes in *jaz5/10* leaves infected with DC3000

JAZs are known to bind a number of transcription factors. Removal of JAZ5 and JAZ10 would be predicted to derepress transcription factors, either constitutively or following pathogen challenge. We compared expression profiles between Col‐0 and *jaz5/10* unchallenged and DC3000‐challenged leaves sampled at 6, 8, 12 and 16 hpi using mRNA‐seq. High‐stringency gFOLD analysis (Feng *et al*., 2012) identified 550 genes with a differential fold change of three or greater between Col‐0 and *jaz5/10* across all time points (Table S3a). At this stringency, *MYC2* and its paralogues *MYC3*,* MYC4* and *AIB1*, implicated in susceptibility to *P. syringae* (Fernandez‐Calvo *et al*., 2011) and insect resistance (Schweizer *et al*., 2013), were not differentially expressed (qRT‐PCR validation; Fig. S6). Neither were the JASMONATE‐ASSOCIATED MYC2‐LIKE1 (JAM1s), recently shown to be negative regulators of jasmonate signalling (Sasaki‐Sekimoto *et al*., 2013). By contrast, the expression of other basic helix‐loop‐helix‐encoding transcription factors, common targets of JAZs, were strongly modified, with seven members being induced in the *jaz5/10* background and 11 being suppressed (Table S4).

Cluster 1, comprising 97 genes suppressed in the *jaz5*/*10* mutant compared with Col‐0 (Fig. S7a), was highly enriched in genes associated with defence responses (Fig. S7b), implicating a positive role for JAZ5/10 in immune defence homeostasis. Apart from the suppression of classical markers of defence, such as *PR5, BGL2* and a chitinase (*CHI*), and an over‐representation of redox components*,* previously characterized defence signalling components implicated in the regulation of SA‐mediated defence were significantly over‐represented. *ALD1*, encoding AGD2‐LIKE DEFENSE RESPONSE PROTEIN 1, is required for the accumulation of SA during *P. syringae* infection, interacting with PAD4 and ICS1 and contributing to the regulation of the abundance and responsiveness of MAMP receptor levels (Cecchini *et al*., 2015). Suppression of *NIMIN1* and *NIMIN2,* whose proteins co‐operate with NPR1 to monitor and appropriately activate defences, such as *PR1* induction (Hermann *et al*., 2013), would compromise SA‐inducible defences, as would the suppression of *AtNUDX6,* encoding a positive regulator of NPR1‐dependent SA signalling (Ishikawa *et al*., 2010).

In parallel, SA‐independent ENHANCED DISEASE SUSCEPTIBILITY1 (EDS1) immunity signalling could be compromised by the suppression of transcripts for the defence regulator flavin‐dependent monooxygenase 1 (*FMO1*; Bartsch *et al*., 2006). FMO is linked to the suppression of cytochrome P450 transcripts *CYP710A1* (Nafisi *et al*., 2007), *CYP71A13* and *PAD3* (Griebel & Zeier, 2010), encoding components of secondary metabolite pathways leading to camalexin and indole‐3‐acetonitrile production, thus implicating weakened broad‐spectrum‐inducible defences in *jaz5/10*.

Of the four *WRKY* transcription factors suppressed in Cluster 1 (*WRKYs 38*,* 51*,* 62* and *75*), WRKY38 and WRKY62 are proposed to be negative regulators of basal defence, interacting with histone deacetylase HDA19 to fine‐tune plant basal defence responses (Kim *et al*., 2008). WRKY62 has been shown to be involved in SA‐mediated suppression of JA signalling downstream of NPR1 (Mao *et al*., 2007), whereas AtWRKY75, in co‐operation with AtWRKY28, transcriptionally regulates SA‐ and JA/ET‐dependent defence signalling pathways (Chen *et al*., 2013).

The impact on the modulation of SA and JA signalling in Cluster 1 is reflected in Cluster 2, comprising 69 genes suppressed in *jaz5/10* leaves at 6 hpi with DC3000 (Fig. S7c) and having an over‐representation of genes associated with the ontological molecular function ‘hydrolase activity’ (Fig. S7d). Most striking is the suppression of JA biosynthetic and signalling components, including *lipoxygenase 2* (*LOX2*), *allene oxide synthase* (*AOS*), *allene oxide cyclase* (*AOC2*) and the JA marker *Vegetative Storage Protein 2* (*VSP2*). Particularly notable was the suppression of *CYP94B3* encoding a JA‐Ile hydroxylase that catalyses the formation of 12‐OH‐JA‐Ile from JA‐Ile (Kitaoka *et al*., 2011). These transcriptional changes are consistent with the unexpected lower JA levels seen in *jaz5/10* (Fig. 4).

## Discussion

This study provides the first demonstration of functional co‐operativity between JAZs. The two phylogenetically distinct JAZs, JAZ5 and JAZ10 (Oh *et al*., 2013), collectively function to mitigate COR virulence functions and contribute to innate immunity.

These findings evolved from unexpected JA dynamics measured in DC3000‐ and DC3000*cor*‐challenged leaves of wild‐type and *coi1*. Contrary to the early expression of marker genes, such as *PDF1.2*, which infer JA biosynthesis (Glazebrook, 2005), JA accumulated very late in the infection process, well after bacterial multiplication and thus incompatible with a direct role in the suppression of SA defences (Robert‐Seilaniantz *et al*., 2011; Pieterse *et al*., 2012; Kazan & Lyons, 2014). Moreover, inoculation with DC3000*cor* further dramatically enhanced this late JA accumulation. These results contrast with the prediction that transcriptional activation of JA signalling and biosynthetic pathways by COR would increase JA levels (Cui *et al*., 2005; Laurie‐Berry *et al*., 2006). Furthermore, *coi1* leaves challenged with DC3000*cor* accumulated markedly more JA than did Col‐0, further indicating a significant COI1‐independent contribution to JA accumulation in DC3000*cor*‐infected leaves. Thus, a complex multilayered defence network or feedback mechanism in response to DC3000 and its phytotoxin COR act to constrain JA accumulation until late in the infection process.

High‐resolution microarray analysis of DC3000‐ and DC3000*hrp*‐infected leaves confirmed the early induction of JA biosynthetic transcripts and a significant, but complex, induction of JAZs in both basal defence and disease progression. A strong, sustained increase in *JAZ*s at 6–7 hpi occurred before, or coincident with, the first detectable trace of COR and paralleled the induction of JA biosynthetic genes. In the absence of early elevated JA levels, we hypothesized that this sustained accumulation of *JAZ*s may restrict jasmonate biosynthesis and signalling. Reduced *JAZ* accumulation reported in leaves challenged with DC3000*cor* may explain the hyperaccumulation of JA (Demianski *et al*., 2012); however, *JAZ5*/*10* dynamics were also MAMP responsive. Counter‐intuitively, despite inducing more JA, DC3000*cor* grows less, suggesting that the early SA increases are epistatic to elevated JA and that JAZs may ameliorate the signalling potential of JA. Although COR alone strongly induced *JAZ5* and *JAZ10*, this induction was transient and required additional co‐stimulatory signals provided by the pathogen. This is resonant of ‘two‐signal’ models which are central to vertebrate immunology and, more recently, have been evoked as mechanisms underpinning aspects of human (Fontana & Vance, 2011) and plant (Lindeberg *et al*., 2012) innate immunity. Moreover, the *JAZ10.3* splice variant accumulated from 8 hpi, indicative of differential splicing during infection and highlighting a further potential mechanism to restrict pathogen modulation of JA signalling (Yan *et al*., 2007; Chung *et al*., 2010). We propose that MAMP triggered immunity primes immunosurveillance and selected JAZ induction and/or COR provide a second discriminatory signal, leading to sustained activation of *JAZ*s and differential splicing of *JAZ10*.

JAZ proteins modulate growth and defence responses through the interplay between JAZ and DELLA proteins (Wild *et al*., 2012; Yang *et al*., 2012) and repress an expanding range of transcription factors involved in various developmental responses (Fernandez‐Calvo *et al*., 2011; Qi *et al*., 2011; Song *et al*., 2011). However, surprisingly little is known about the extent to which individual JAZs collaborate to regulate specific biological processes. Using reverse genetic approaches, we identified unexpected JAZ co‐operativity. Single loss‐of‐function mutations in all tested *jaz* alleles did not significantly modify DC3000 infection phenotypes. However, the *jaz5*/*10* double mutant, but no other combinations, including those with *jaz5* or *jaz10*, resulted in remarkably strong chlorosis. *jaz5*/*10* lines were moderately more susceptible to DC3000, albeit weakly so compared with the hypersusceptible *pad4* or *sid2*. In root growth assays, *jaz5/10* mutants showed similar sensitivity to MeJA and COR as other double *jaz* mutants, but chlorosis was conditional on the production of COR. Co‐infiltration of DC3000*cor* with COR, but not MeJA or JA‐Ile, could induce strong chlorosis in *jaz5/10*. In addition, HopX delivery could not induce chlorosis in *jaz5/10* leaves.

Although *MYC2*,* MYC3* and *MYC4* are required for full susceptibility to DC3000 (Nickstadt *et al*., 2004; Laurie‐Berry *et al*., 2006; Fernandez‐Calvo *et al*., 2011), no sustained induction was evident in challenged or unchallenged *jaz5*/*jaz10* leaves. DC3000‐induced chlorosis was not enhanced in *myc2*/*jaz5*/*jaz10* leaves and was minimal in *MYC2* overexpression lines. Thus, other transcription inducers or repressors (Wild *et al*., 2012; Yang *et al*., 2012), tethered by JAZ5/10, probably contribute to COR‐induced chlorosis. Indeed, mRNA‐seq profiling revealed that *jaz5*/*10* plants had significantly fewer transcripts associated with defence responses, particularly SA‐mediated processes, than Col‐0. This was reflected in strongly suppressed JA biosynthesis and signalling transcripts in *jaz5/*10 mutants within 6 h of DC3000 infection, helping to explain why the anticipated increase in JA was not measured in these plants.

JA responses are fine‐tuned through a number of JAZ interactions, including homo‐ and heterodimerization, concentration‐ and ligand‐dependent differential binding to COI1, and direct or indirect interaction with transcriptional repressors, such as TOPLESS (Pauwels *et al*., 2010). For example, JAZ8 is susceptible to *in vivo* 26S proteasome degradation by COR, but more resistant to JA‐mediated degradation than is JAZ10 (Shyu *et al*., 2012). Moreover, there are COI1‐independent jasmonate responses, suggesting that other F‐Box proteins are involved in jasmonate signalling (Geng *et al*., 2012). Although biotic challenge leads to transcriptional changes in *JAZ*s, no direct role for JAZs in plant–pathogen interactions has yet been clearly established. One‐quarter of transgenic lines overexpressing *JAZ1* lacking the Jas domain phenocopied *coi1* mutants show JA insensitivity and increased resistance to DC3000 infection (Thines *et al*., 2007). A similar proportion of plants overexpressing JAZ1^A205A206^, which disrupts the JA‐Ile/COR‐dependent COI1–JAZ interaction, also conferred JA insensitivity, some of which additionally showed increased resistance to DC3000 infection (Shyu *et al*., 2012). Thus, the overexpression of dominant negatives can interfere with DC3000 proliferation; however, no evidence for altered susceptibility to *jaz1* has been shown. Similarly, *jaz10* antisense lines showed moderately enhanced symptom development, but not increased susceptibility to DC3000 (Demianski *et al*., 2012). We found that *jaz5*/*10* chlorosis was specific to exogenous COR, but not JA‐Ile or MeJA. JAZ5 can be directly or indirectly involved in transcriptional repression through either its two EAR domains (Kagale *et al*., 2010) or via its interaction with the negative regulators TOPLESS (Arabidopsis Interactome Mapping Consortium, 2011; Causier *et al*., 2012) and NINJA (Pauwels *et al*., 2010). Furthermore, JAZ5 lacks the highly conserved LPIARR motif found in many JAZ proteins that interact strongly with COI1 in a JA‐Ile‐dependent manner (Shyu *et al*., 2012). JAZ5 could be eliminated via a COI1‐independent mechanism, or require molecules other than JA‐Ile for its degradation. JAZ10 could also function as a negative regulator of JA responses. *jaz10* loss‐of‐function mutants show JA hypersensitivity (Yan *et al*., 2007). Thus, both JAZ5 and JAZ10 could act as negative regulators of a broad range of JA responses, with COR being particularly effective at activating these responses.

COR‐producing *P. syringae* induces strong chlorosis on tomato, but not *Arabidopsis*, leaves. COR‐induced cell death in tomato is via the modulation of the photosynthetic machinery and/or reactive oxygen signalling (Ishiga *et al*., 2009). DC3000 infection suppressed *chloroplast peroxiredoxin* (*Prx*) and *NADPH‐dependent thioredoxin reductase C* (*NTRC*) in tomato and *Arabidopsis*, but COR only suppressed *Prx* and *NTRC* in tomato (Ishiga *et al*., 2012). Neither chloroplast *Prx* nor *NTRC* were differentially regulated in our mRNA‐seq experiment. Tomato SlJAZ2, SlJAZ6 and SlJAZ7 proteins interact with SlCOI1 in the presence of COR. However, silencing of these genes in tomato and *Nicotiana benthamiana* led to no significant differences in COR‐induced chlorosis or enhanced bacterial multiplication (Ishiga *et al*., 2013), suggesting that additional pathogen‐associated factors are required for COR virulence in *Arabidopsis*, as inferred by the *JAZ5*/*10* dynamics observed in this study.

In summary, our data reveal unexpected JA dynamics during DC3000 infection of *Arabidopsis* and co‐operativity between JAZ5 and JAZ10 which restricts COR phytotoxicity. This co‐operativity is remarkable as it protects against a pathogen virulence factor that has evolved as a plant hormone mimic. The future challenge is to understand the complex regulatory network of JAZ proteins, the biological relevance of JAZ–JAZ homo‐ and heterodimers and the identity of the interacting transcription factors/repressors involved in this particular biological process.

## Author contributions

M.T.Z., B.Z., W.T., S.T. and M.G. designed the research. M.T.Z., B.Z., G.E. and R.W. performed the experiments. M.T.Z., B.Z., S.J., R.Y., N.S. and M.G. analysed the data. B.Z., G.E., R.W. and W.T. collected material. M.T.Z., S.J. and M.G. interpreted the data. M.G., N.S. and M.T.Z. wrote the manuscript.

## Supporting information

Please note: Wiley Blackwell are not responsible for the content or functionality of any supporting information supplied by the authors. Any queries (other than missing material) should be directed to the *New Phytologist* Central Office.

**Fig. S1** Jasmonic acid (JA) accumulates late in leaves infected with virulent DC3000.
**Fig. S2** The majority of DC3000‐induced jasmonic acid (JA) is derived from *de novo* JA biosynthesis.
**Fig. S3** Reverse transcription‐polymerase chain reaction (RT‐PCR) validation of *jaz5*, j*az10* and *jaz5/10* knock‐out lines.
**Fig. S4** Jasmonate sensitivity to seedling root growth of different double *jaz* mutant combinations compared with the jasmonate insensitive *coi1‐*16.
**Fig. S5** The *jaz5/10* chlorotic phenotype is not dependent on MYC2.
**Fig. S6** Expression profiles of four JAZ‐targeted *MYC* genes in wild‐type and *jaz5/10* mutant backgrounds.
**Fig. S7** Gene clusters discriminating *jaz5/10* and Col‐0 plants in non‐induced leaves or early after leaf infection with DC3000.
**Table S1** Summary of pathogen infection phenotypes of *JAZ* T‐DNA insertion lines, including response of double and triple mutant combinations used in this study
**Table S2** *JAZ* and jasmonate biosynthetic gene expression derived from CATMA arrays, reporting a compatible interaction, a basal defence response or the impact of DC3000 type III effectors
**Methods S1** Primers used for reverse transcription‐polymerase chain reaction (RT‐PCR) and their respective amplicon size.

**Table S3** Differential gene expression between Col‐0 and *jaz5/10* mutants as determined by gFOLD, and selected gene clusters derived from gFOLD analysis discriminating Col‐0 from *jaz5/10* mutants

**Table S4** Basic helix‐loop‐helix transcription factor genes differentially regulated between wild‐type Col‐0 challenge and the *jaz5/10* mutant following infection
