# Supplementary material for: Novel JAZ co‐operativity and unexpected JA dynamics underpin Arabidopsis defence responses to Pseudomonas syringae infection
Source: New Phytol. 2015 Oct 2;209(3):1120–34. doi: 10.1111/nph.13683 (PMC4791170; doi:10.1111/nph.13683)
Supplement: Supplementary file 1 — Fig. S1 Jasmonic acid (JA) accumulates late in leaves infected with virulent DC3000. Fig. S2 The majority of DC3000‐induced jasmonic acid (JA) is derived from de novo JA biosynthesis. Fig. S3 Reverse transcription‐polymerase chain reaction (RT‐PCR) validation of jaz5, jaz10 and jaz5/10 knock‐out lines. Fig. S4 Jasmonate sensitivity to seedling root growth of different double jaz mutant combinations compared with the jasmonate insensitive coi1‐16. Fig. S5 The jaz5/10 chlorotic phenotype is not dependent on MYC2. Fig. S6 Expression profiles of four JAZ‐targeted MYC genes in wild‐type and jaz5/10 mutant backgrounds. Fig. S7 Gene clusters discriminating jaz5/10 and Col‐0 plants in non‐induced leaves or early after leaf infection with DC3000. Table S1 Summary of pathogen infection phenotypes of JAZ T‐DNA insertion lines, including response of double and triple mutant combinations used in this study Table S2 JAZ and jasmonate biosynthetic gene expression derived from CATMA arrays, reporting a compatible interaction, a basal defence response or the impact of DC3000 type III effectors Methods S1 Primers used for reverse transcription‐polymerase chain reaction (RT‐PCR) and their respective amplicon size. [file NPH-209-1120-s001.pdf]

New Phytologist

New Phytologist Supporting Information Figs S1–S7, Tables S1 & S2 and Methods S1

Article title: Novel JAZ co-operativity and unexpected JA dynamics underpin Arabidopsis defence responses to *Pseudomonas syringae* infection

Authors: Marta de Torres Zabala, Bing Zhai, Siddharth Jayaraman, Garoufalia Eleftheriadou, Rebecca Winsbury, Ron Yang, William Truman, Saijung Tang, Nicholas Smirnoff and Murray Grant

Article acceptance date: 19 August 2015

The following Supporting Information is available for this article:

Fig. S1 JA accumulates late in leaves infected with virulent DC3000.
Fig. S2 The majority of DC3000 induced JA is derived from *de novo* JA biosynthesis.
Fig. S3 Reverse transcriptase PCR validation of *jaz5*, j*az10* and *jaz5/10* knock-out lines.
Fig. S4 Jasmonate sensitivity to seedling root growth of different double *jaz* mutant combinations compared to the jasmonate insensitive *coi1*-16.
Fig. S5 The *jaz5/10* chlorotic phenotype is not dependent upon MYC2.
Fig. S6 Expression profiles of four JAZ targeted *MYC* genes in wild type and *jaz5/10* mutant backgrounds.
Fig. S7 Gene clusters discriminating *jaz5/10* and Col-0 plants in noninduced leaves or early after leaf infection with DC3000.
Table S1 Summary of pathogen infection phenotypes of *JAZ* T-DNA insertion lines, including response of double and triple mutant combinations used in this study
Table S2 *JAZ* and jasmonate biosynthetic gene expression derived from CATMA arrays reporting a compatible interaction, a basal defense response or the impact of DC3000 type III effectors
Table S3 Differential gene expression between Col-0 and *jaz5/10* mutants as determined by G-fold and selected gene Clusters derived from gFOLD analysis discriminating Col-0 from *jaz5/10* mutants (separate Excel file)
Table S4 Basic helix loop helix transcription factors genes differentially regulated between wild type Col-0 challenge and the *jaz5/10* mutant following infection (separate Excel file)
Methods S1 Primers used for RT-PCR and their respective amplicon size.

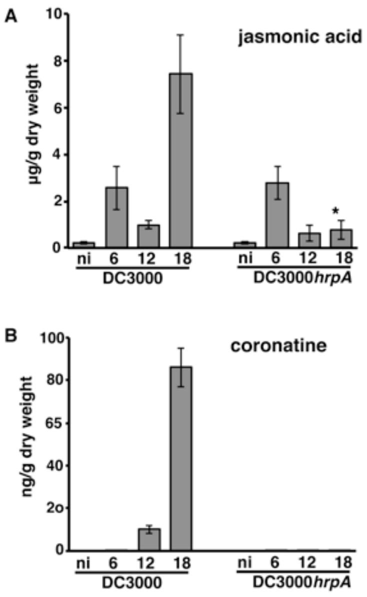

Fig. S1 JA accumulates late in *A. thaliana* leaves infected with virulent *P. syringae* DC3000. (a) Following an initial wound response JA accumulates late in the infection process, whereas leaves challenged with the DC3000*hrpA* mutant show only the initial wound response and do not accumulate appreciable amounts of COR relative to noninoculated tissue at 18hpi.(b) In the compatible interaction, COR is detectable 6 hpi and accumulates rapidly to 16 hpi, whereas only a very small amount of coronatine is detectable following challenge with the DC3000*hrpA* mutant*.* Time in hpi, ni = noninduced. Significant difference (*t*-test *, $P < 0.01$) between corresponding time-points ($n = 4$, mean ± SD). Figure representative of three independent experiments.

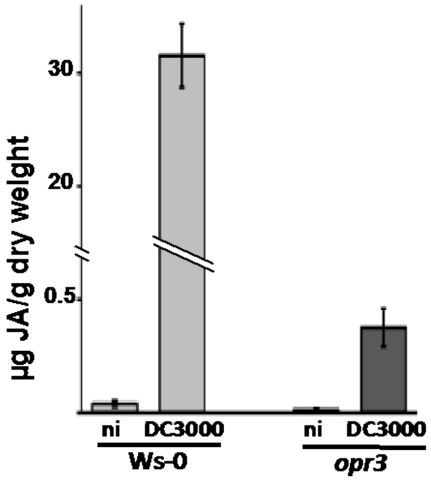

Fig. S2 The majority of *P. syringae* DC3000 induced JA is derived from *de novo* JA biosynthesis. JA accumulates strongly 20 hpi in DC3000 challenged *A. thaliana* leaves of accession Ws-0, whereas *opr3* mutants lacking *2-oxophytodienoic acid reductase* have *c*. 80-fold lower JA levels (*n* = 3, mean ± SD). Data are representative of two independent experiments.

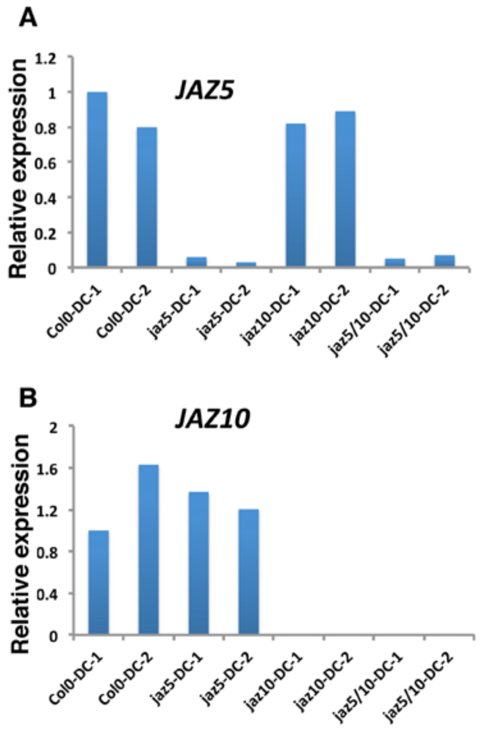

Fig. S3 RT-PCR validation of *A. thaliana jaz5*, *jaz10* and *jaz5/10* mutant lines. To determine the impact of the specific T-DNA insertions, *P. syringae* DC3000 challenge (OD_{600} 0.15) was used to induced *JAZ* genes in Col-0 or *jaz* mutants. Duplicate samples harvested 8 hpi and total RNA extraction and qRT-PCR were performed as described (de Torres Zabala *et al.*, 2009). *Actin 2* (At3g18780) was used as internal standard to normalize cDNA abundance between samples. Relative expression levels are expressed in arbitrary units using a *P. syringae* DC3000 challenged Col-0 replicate as being equivalent to 1. (a) Relative expression of *JAZ5* in *jaz5*, *jaz10* and *jaz5/10* mutant backgrounds 8 hpi with DC3000. (b) Relative expression of *JAZ10* in *jaz5*, *jaz10* and *jaz5/10* mutant backgrounds 8 hpi with DC3000. See Methods S1 for RT-PCR primer sequences.

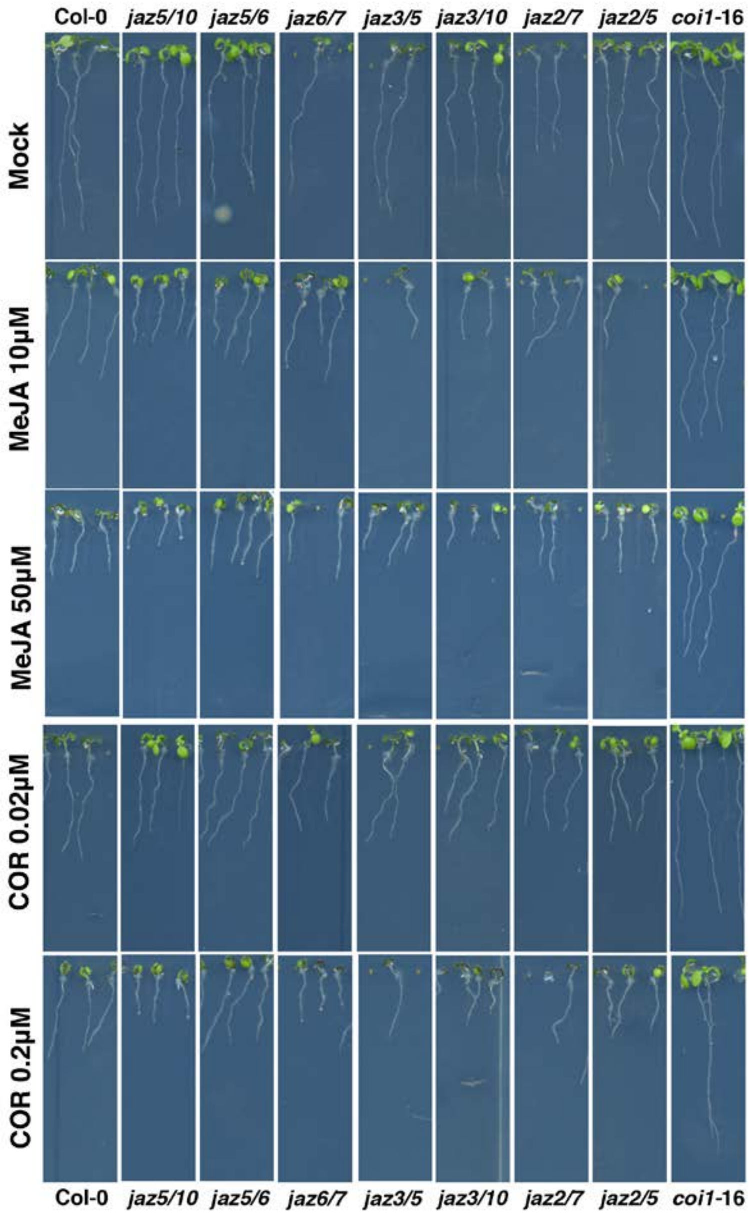

Fig. S4 Jasmonate sensitivity to seedling root growth of various *A. thaliana* double *jaz* mutant combinations compared to *coi1*-16 on media supplemented with MeJA (10 or 50 µM) or COR (0.02 or 0.2 µM) and photographed 8 d later. Importantly, *jaz5/10* mutants were not noticeably different from other tested double mutant combinations with respect to their response to either MeJA or COR.

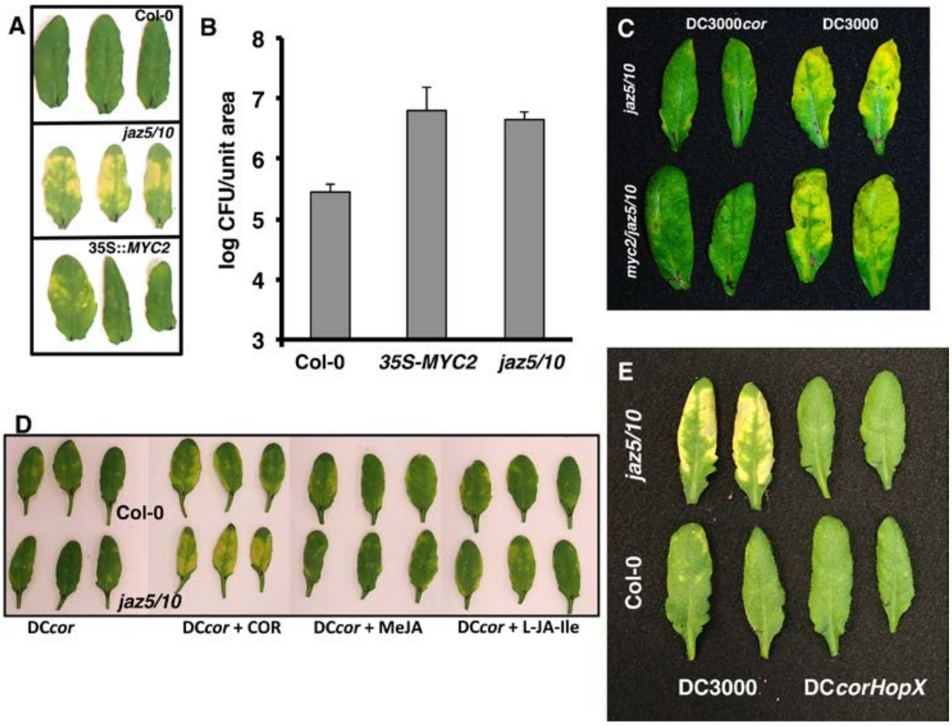

Fig. S5 The *A. thaliana jaz5/10* chlorotic phenotype is not dependent upon MYC2. (a) *jaz5/10,* but not wild type Col-0 or 35S*::MYC2* overexpression lines display characteristic strong chlorotic symptoms 3 dpi with *P. syringae* DC3000 (OD_{600} 0.001). (b) Despite differences in extent of chlorosis, both *jaz5/10* and 35S*::MYC2* plant support enhanced growth of DC3000 multiplication 4 dpi (OD_{600} 0.0002; $n = 4$, mean ± SD). This experiment was repeated twice with similar results. (c) Introducing the *myc2* mutation into the *jaz5/10* background did not modify the *jaz5/10* phenotype following DC3000 challenge (OD_{600} 0.001). Photograph taken 4 dpi. (d) The *jaz5/10* chlorotic phenotypes are specific to COR. Leaves of Col-0 or *jaz5/10* were co-infiltrated with DC3000Δ*cfa6:*Δ*cmaA* (OD_{600} 0.002) and COR (0.2µM), MeJA (50mM) or JA-L-Ile (50mM) and then 2 d later with the same jasmonate concentration. Leaves were photographed 4 dpi. (e) Delivery of HopX does not mimic coronatine and induce strong chlorotic symptoms in *jaz5/10* plants. Col-0 and *jaz5/10* plants were challenged with DC3000 or DC3000*cor* carrying *HopX* (Gimenez-Ibanez *et al.*, 2014).

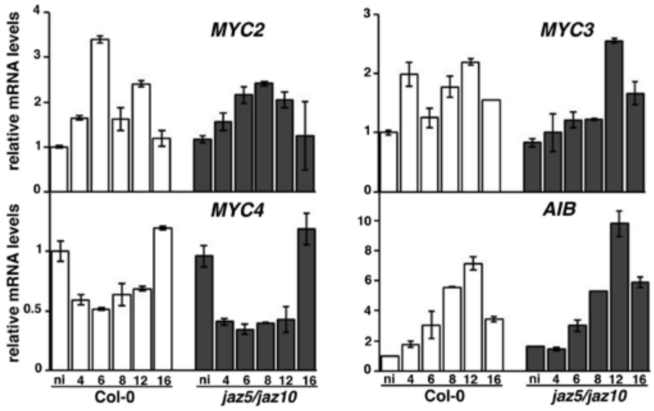

Fig. S6 Expression profiles of four JAZ targeted *MYC* genes in wild type and *jaz5/10* mutant *A. thaliana* backgrounds. No major changes in the expression of the genes encoding JAZ targets, *MYC2*,*3*,*4* and *AIB1* genes (Fernandez-Calvo *et al*., 2011) between wild-type Col-0 and the *jaz5/10* mutant were evident following challenge with *P. syringae* DC3000 as would be anticipated if dynamically involved in JAZ5/10's role in protecting against COR cytotoxicity. Relative expression levels are expressed in arbitrary units using the unchallenged (ni, noninduced) Col-0 samples as being equivalent to 1. Error bars represent ± SD of three technical replicates from pooled biological replicates, and *x*-axis values represent time in hours post inoculation.

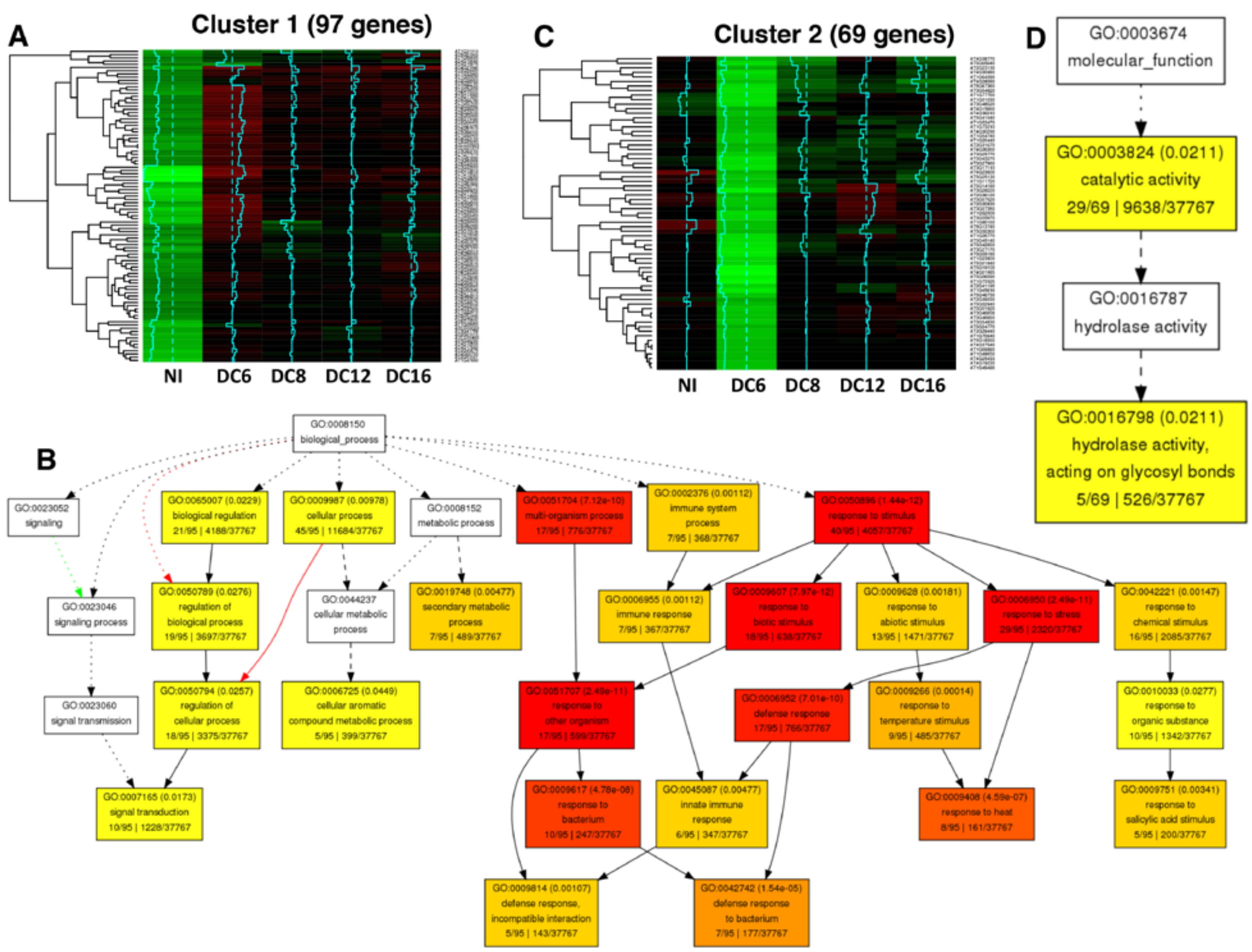

Fig. S7 Clusters discriminating *A. thaliana jaz5/10* and Col-0 plants in noninduced leaves and during early responses to *P. syringae* DC3000 infected leaves. Clusters (Table S3b) were generated from gFOLD outputs using Dynamic Tree Cut which is a dynamic branch cutting algorithm for detecting clusters in a cluster tree based on their shape (http://labs.genetics.ucla.edu/horvath/CoexpressionNetwork/BranchCutting/Supplement.pdf). (a) Cluster 1 highlights genes which are suppressed in unchallenged *jaz5/10* leaves relative to Col-0 control plants. AgriGO gene ontology analysis (b) revealed a highly significant enrichment of genes with biological process terms associated with defense responses to bacteria, response to incompatible interactions, response to heat, signal transduction and SA signaling in this cluster (http://bioinfo.cau.edu.cn/agriGO/analysis.php). Cluster 2 (c) comprises genes significantly repressed in *jaz5/10* 6h after challenge with DC3000 and is overrepresented in genes aligned to the Molecular Function term ‘hydrolase activity’ (d).

New Phytologist

Table S1 Summary of *JAZ* T-DNA insertion lines, including double and triple mutant combinations used in this study

Name	SALK ID	Phenotype (leaf chlorosis)
Single		
jaz1 (At1g19180)	SALK_011957	0
jaz2 (At1g74950)	SALK_025279	0
jaz3 (At3g17860)	SALK_139337	0
jaz4 (At1g48500)	SALK_026633	0
jaz5 (At1g17380)	SALK_053775	0
jaz6 (At1g72450)	SAIL_1156_C06	0
jaz7 (At2g34600)	WiscDsLox7H11	0
jaz10 (At5g13220)	SAIL_92_D08	X
jaz12 (At5g20900)	SALK_055032	0
Double		
jaz1/3		0
jaz2/5		0
jaz2/7		0
jaz3/5		0
jaz3/10		X
jaz5/6		0
jaz6/7		0
jaz5/7		0
jaz5/10		XXX
jaz7/10		X
Triple		
jaz 2/5/10		XXX
jaz 3/5/10		XXX
jaz 6/5/10		XXX
jaz 7/5/10		XXX

AGI code and provenance of single T-DNA insertion lines is shown. Right hand column records corresponding infection phenotype following challenge with *P. syringae* pv. tomato strain DC3000. Degree of chlorosis is represented on a scale of 0 to XXX, where 0 indicates no difference in comparison to the wild type control. See Fig. 3 for strong chlorotic symptoms (XXX). See Fig. S3 for RT-PCR validation of *jaz5/10* double mutant.

Table S2 Expression values from CATMA arrays (four biological replicated and two technical replicates (see Breeze *et al*., 2011, for array design and data extraction details) corresponding to the mean (log_2) fold change observed between the treatments reporting respectively, a compatible interaction (DC3000 vs $MgCl_2$), basal defense response (DC3000*hrpA* vs. $MgCl_2$) and DC3000 vs. DC3000*hrpA* (impact of type III effectors)

Supplementary Table 1 - de Torres et al. Data corresponding to Figure 2

**Figure 2A (i)**															
	**DC3000 vs. $MgCl_2$**														
Gene	AGI	CATMA_C	0	2	3	4	6	7	8	10	11	12	14	16	17.5
AOC4	AT1G13280	CATMA1a12280	0.039828	0.62853	0.18513	1.0427	0.029465	-0.23968	-0.29439	-0.26207	-0.06638	0.55064	0.27401	0.63044	0.81537
LOX3	AT1G17420	CATMA1a16465	0.091227	-1.0382	0.00426	-0.76523	-1.0024	-1.711	-1.4043	-2.4297	-1.9246	-2.8421	-2.827	-3.0118	-2.6673
OPCL1	AT1G20510	CATMA1a19510	-0.23465	-0.47501	-0.29654	-0.80107	-1.6411	-1.6527	-1.4839	-1.4255	-0.95162	-1.2435	-1.3436	-1.3921	-1.3002
LOX4	AT1G72520	CATMA1a61750	0.00338	-1.0123	-0.00108	-0.98	-1.2991	-1.8338	-1.552	-2.3853	-1.7154	-2.8367	-2.576	-2.4742	-2.7581
OPR3	AT2G06050	CATMA2a04825	-0.05545	-0.21191	0.54072	0.036895	-0.92898	-1.3717	-1.3022	-1.6726	-0.7431	-1.797	-1.6639	-1.3647	-1.6481
AOC3	AT3G25780	CATMA3c57389	0.021517	-0.91417	-0.22266	-0.88946	-1.7692	-2.5651	-2.2984	-3.0804	-2.3389	-3.4154	-3.2747	-2.604	-2.6272
CYP74B2	AT4G15440	CATMA4a16160	-0.05571	1.1161	1.0021	1.8207	0.87894	0.91326	1.1014	1.1106	1.058	1.0105	0.95968	0.77858	0.90386
**Figure 2A (ii)**															
	**DC3000*hrpA* vs. $MgCl_2$**														
AOC4	AT1G13280	CATMA1a12280	0.53936	0.43346	0.59244	0.89235	0.74446	0.79755	0.47585	1.1124	0.4601	0.85357	0.86506	0.34651	0.45157
LOX3	AT1G17420	CATMA1a16465	0.049852	-0.36519	-0.55921	-0.7203	-0.71351	-0.8988	-1.0201	-0.95739	-0.83853	-1.6639	-1.8262	-1.7104	-1.0742
OPCL1	AT1G20510	CATMA1a19510	-0.34601	-0.24815	-0.86353	-0.67293	-1.0001	-0.9124	-1.0026	-0.86716	-0.54756	-0.92602	-0.90726	-1.1042	-0.77969
LOX4	AT1G72520	CATMA1a61750	-0.26019	-0.96979	-0.87553	-0.8125	-1.1809	-1.3089	-1.4686	-1.4086	-0.70843	-1.4855	-1.8696	-1.3453	-1.2314
OPR3	AT2G06050	CATMA2a04825	-0.05983	0.014886	0.02472	-0.41151	-0.4899	-0.51622	-0.77801	-0.47853	0.036183	-0.68899	-0.67151	-0.63435	-0.70117
AOC3	AT3G25780	CATMA3c57389	-0.22573	-0.53986	-0.17935	-0.85758	-1.2631	-1.1104	-1.7172	-1.6745	-0.786	-2.0572	-2.17	-1.3861	-1.1999
CYP74B2	AT4G15440	CATMA4a16160	0.18555	1.0825	1.3989	1.4352	1.4972	1.7256	1.3738	1.9332	1.1832	1.293	1.0108	0.49178	0.66297
**Figure 2A (iii)**															
	**DC3000 vs. DC3000*hrpA***														
AOC4	AT1G13280	CATMA1a12280	-0.49953	0.19507	-0.40731	0.15034	-0.71499	-1.0372	-0.77023	-1.3745	-0.52648	-0.30293	-0.59105	0.28393	0.3638
LOX3	AT1G17420	CATMA1a16465	0.041376	-0.67296	0.56347	-0.04493	-0.28885	-0.81223	-0.38414	-1.4723	-1.0861	-1.1783	-1.0007	-1.3015	-1.5931
OPCL1	AT1G20510	CATMA1a19510	0.11135	-0.22686	0.56699	-0.12814	-0.64099	-0.74031	-0.48129	-0.55836	-0.40406	-0.31746	-0.43633	-0.28783	-0.52051
LOX4	AT1G72520	CATMA1a61750	0.26357	-0.04255	0.87444	-0.1675	-0.11816	-0.52489	-0.08339	-0.97665	-1.007	-1.3512	-0.70644	-1.1289	-1.5266
OPR3	AT2G06050	CATMA2a04825	0.004381	-0.2268	0.516	0.44841	-0.43908	-0.85544	-0.52415	-1.1941	-0.77928	-1.108	-0.99235	-0.73033	-0.94689
AOC3	AT3G25780	CATMA3c57389	0.24725	-0.37431	-0.0433	-0.03188	-0.50608	-1.4547	-0.58128	-1.4059	-1.5529	-1.3582	-1.1047	-1.2179	-1.4273
CYP74B2	AT4G15440	CATMA4a16160	-0.24126	0.033591	-0.39677	0.38549	-0.61827	-0.81238	-0.27242	-0.82259	-0.12517	-0.28255	-0.05109	0.2868	0.24089
**Figure 2B (i)**															
	**DC3000 vs. $MgCl_2$**														
Gene	AGI	CATMA_C	0	2	3	4	6	7	8	10	11	12	14	16	17.5
JAZ1	At1g19180	CATMA1a18220	-0.31462	-0.53278	-0.10801	-0.9539	-1.8923	-2.1991	-2.1225	-2.7374	-2.1371	-3.3765	-2.7294	-2.7745	-3.2012
JAZ2	At1g74950	CATMA1a64320	-0.19274	-0.5085	0.076901	0.27589	-1.9813	-1.7799	-1.9238	-1.7773	-1.2438	-2.3675	-2.328	-1.8569	-2.1377
JAZ3	At3g17860	CATMA3a17360	-0.06525	0.19409	0.43872	0.27482	-1.0535	-1.191	-1.0892	-1.0302	-0.81897	-1.0912	-1.113	-0.60966	-0.56445
JAZ4	At1g48500	CATMA1a39600	-0.10411	-0.09793	0.16517	0.31026	-0.02688	-0.11488	0.28041	0.29837	0.30664	0.36596	-0.16356	0.1757	0.41936
JAZ5	At1g17380	CATMA1a16410_new	-0.2354	-0.66286	-0.10439	-0.56688	-1.8627	-1.7538	-1.676	-1.9553	-1.1641	-2.2983	-1.877	-1.7602	-1.6434
JAZ6	At1g72450	CATMA1a61670	-0.36064	-0.1014	0.16567	-0.35452	-1.504	-1.5511	-1.5396	-1.4804	-0.91011	-2.0571	-1.4254	-1.3521	-1.1964
JAZ7	At2g34600	CATMA2a32730	-0.23435	0.072871	0.032922	-0.37928	-1.2196	-1.6228	-1.8575	-2.0174	-1.4376	-2.7316	-2.098	-1.9869	-2.1797
JAZ8	At1g30135	CATMA1c71469	-0.12966	-0.99934	-0.45481	-0.56525	-1.448	-1.3319	-2.0777	-1.9901	-1.9414	-2.4322	-2.6051	-2.5382	-2.3395
JAZ9	At1g70700	CATMA1a59980	-0.31347	0.053491	0.2466	0.28333	-1.9441	-2.0126	-1.772	-1.9395	-1.2728	-2.2172	-2.1018	-1.6615	-1.6647
JAZ10	At5g13220	CATMA5a11430	-0.54939	-0.49449	-0.16092	-0.48977	-2.6402	-2.8738	-3.3041	-3.5151	-3.0166	-4.1866	-3.8718	-3.2447	-3.5325
JAZ11	At3g43440	CATMA3a35920	-0.01751	0.007395	0.046892	0.20594	-0.2559	-0.21947	-0.64038	-0.921	-1.2442	-1.3551	-1.4695	-1.151	-1.401
JAZ12	At5g20900	CATMA5a19440	0.042422	0.008617	0.16341	-0.04674	-1.0442	-0.54856	-0.43536	-0.3339	-0.21972	-0.71865	-0.49779	-0.49645	-0.5132
AtMYC2	At1g32640	CATMA1a31015	-0.18355	0.053432	0.30426	0.15539	-1.7995	-1.7799	-1.251	-1.1462	-0.62169	-1.2817	-0.93915	-0.44192	-0.40431
**Figure 2B (ii)**															
	**DC3000*hrpA* vs. $MgCl_2$**														
JAZ1	At1g19180	CATMA1a18220	-0.02477	-0.47068	-0.42241	-0.9615	-1.649	-1.4585	-2.0774	-2.0328	-1.2142	-2.3076	-2.3162	-1.5622	-1.8489
JAZ2	At1g74950	CATMA1a64320	-0.13277	0.25716	-0.28679	-0.3757	-1.0547	-0.82184	-1.1136	-0.47336	-0.32074	-0.84678	-1.0258	-0.83096	-0.69328
JAZ3	At3g17860	CATMA3a17360	0.2742	0.29888	0.24751	-0.11623	-0.02137	-0.0717	-0.27156	0.24816	0.12115	-0.24393	-0.25812	-0.11206	0.03511
JAZ4	At1g48500	CATMA1a39600	-0.32235	0.076419	0.25916	0.064912	-0.14086	0.19738	0.15664	0.21173	0.27679	0.31136	0.24285	0.38273	0.43534
JAZ5	At1g17380	CATMA1a16410_new	0.035033	-0.37315	-0.52187	-0.66707	-1.1317	-0.8504	-1.1497	-0.99042	-0.60957	-1.2681	-1.059	-0.87125	-0.84563
JAZ6	At1g72450	CATMA1a61670	0.19627	0.068353	-0.19256	-0.67218	-1.0437	-0.95826	-1.1769	-0.7709	-0.201	-0.89567	-0.74166	-0.47146	-0.09286
JAZ7	At2g34600	CATMA2a32730	0.18397	0.20909	0.23011	-0.18233	-0.36464	-0.02972	-0.70296	-0.15622	-0.00529	-0.51073	-0.57571	-0.47466	-0.34651
JAZ8	At1g30135	CATMA1c71469	-0.16591	-0.42144	-0.54083	-0.51583	-0.97431	-0.84674	-1.4877	-1.0534	-1.1805	-1.1169	-1.8866	-1.1215	-0.6954
JAZ9	At1g70700	CATMA1a59980	0.093986	0.26572	-0.10381	-0.20663	-0.58388	-0.38723	-0.59965	-0.02944	0.032532	-0.39755	-0.56918	-0.51206	-0.29329
JAZ10	At5g13220	CATMA5a11430	0.23502	-0.11776	-0.25382	-0.63079	-1.3255	-0.89416	-1.5456	-1.273	-1.312	-2.0076	-2.1483	-1.3922	-1.2059
JAZ11	At3g43440	CATMA3a35920	0.003639	-0.04047	0.45054	0.14175	0.073052	0.062659	-0.18826	-0.29357	-0.66746	-0.46193	-0.75071	-0.49492	-0.32749
JAZ12	At5g20900	CATMA5a19440	-0.10559	-0.02121	0.13755	-0.26302	-0.2138	-0.04086	-0.27229	0.032556	0.14651	-0.21002	-0.4581	-0.46477	-0.23343
AtMYC2	At1g32640	CATMA1a31015	-0.0658	0.26548	-0.06986	0.177	-0.06167	-0.20904	-0.0012	0.36206	0.30542	-0.16814	-0.02316	-0.29203	0.038485
**Figure 2B (iii)**															
	**DC3000 vs. DC3000*hrpA***														
JAZ1	At1g19180	CATMA1a18220	-0.28985	-0.0621	0.3144	0.007598	-0.24328	-0.7406	-0.04507	-0.70458	-0.92289	-1.0689	-0.41311	-1.2123	-1.3523
JAZ2	At1g74950	CATMA1a64320	-0.05997	-0.76566	0.36369	0.65158	-0.92653	-0.95803	-0.81027	-1.304	-0.92308	-1.5207	-1.3022	-1.0259	-1.4444
JAZ3	At3g17860	CATMA3a17360	-0.33945	-0.10479	0.19121	0.39105	-1.0321	-1.1193	-0.81767	-1.2783	-0.94012	-0.84722	-0.85483	-0.49759	-0.59956
JAZ4	At1g48500	CATMA1a39600	0.21823	-0.17434	-0.09398	0.24535	0.11398	-0.31226	0.12376	0.086634	0.029853	0.054605	-0.40641	-0.20703	-0.01598
JAZ5	At1g17380	CATMA1a16410_new	-0.27043	-0.28971	0.41749	0.10019	-0.73102	-0.90335	-0.52633	-0.9649	-0.55457	-1.0302	-0.81799	-0.88894	-0.79776
JAZ6	At1g72450	CATMA1a61670	-0.55691	-0.16975	0.35823	0.31766	-0.46024	-0.5928	-0.36267	-0.70948	-0.70911	-1.1614	-0.68372	-0.88064	-1.1035
JAZ7	At2g34600	CATMA2a32730	-0.41832	-0.13622	-0.19719	-0.19695	-0.855	-1.5931	-1.1546	-1.8611	-1.4324	-2.2209	-1.5223	-1.5123	-1.8332
JAZ8	At1g30135	CATMA1c71469	0.03625	-0.57791	0.086028	-0.04942	-0.47365	-0.48517	-0.59	-0.93671	-0.76097	-1.3153	-0.71851	-1.4167	-1.6441
JAZ9	At1g70700	CATMA1a59980	-0.40746	-0.21223	0.3504	0.48996	-1.3602	-1.6254	-1.1723	-1.9101	-1.3053	-1.8197	-1.5327	-1.1494	-1.3714
JAZ10	At5g13220	CATMA5a11430	-0.78441	-0.37673	0.092902	0.14102	-1.3147	-1.9796	-1.7584	-2.2421	-1.7046	-2.179	-1.7236	-1.8525	-2.3267
JAZ11	At3g43440	CATMA3a35920	-0.02115	0.04786	-0.40365	0.064184	-0.32895	-0.28213	-0.45212	-0.62743	-0.57673	-0.89313	-0.71883	-0.65607	-1.0736
JAZ12	At5g20900	CATMA5a19440	0.14801	0.029824	0.025864	0.21627	-0.8304	-0.5077	-0.16307	-0.36645	-0.36623	-0.50863	-0.0397	-0.03168	-0.27977
AtMYC2	At1g32640	CATMA1a31015	-0.11775	-0.21205	0.37412	-0.02161	-1.7378	-1.5709	-1.2498	-1.5083	-0.9271	-1.1135	-0.91599	-0.14989	-0.4428

These data were used to generate the heat maps in Fig. 2 based upon a *t*-test significance cut-off at $P \leq 0.01$. Raw data is available from GEO (Gene Expression Omnibus) under the accession number GSE56094.

New Phytologist

Methods S1 Primers used to obtain the cDNA standards for RT-PCR and associated amplicon size.

ACTIN2: F 5’-CATGTTTGAGACCTTTAACTCTCC -3’; R 5’-AACGATTCCTGGACCTGCCTCATC-3’, 744bp.
JAZ5: F 5’-ATGTCGTCGA GCAATGAAAATGC-3’; R 5’-GCAAAGAATCGATGGAGGGAAG-3’, 578bp.
JAZ10 (10.1 and 10.3): F 5’-ATGTCGAAAGCTACCATAGAACTC-3’; R 5’-CTCCTTCTAGATTCTGGCCAAAG-3’, 496bp.
JAZ10 (10.1,10.3 and 10.4): F 5’-ATGTCGAAAGCTACCATAGAACTC-3’; R 5’-GCCGATGTCGGATAGTAAGGAG -3’, 567bp, 624bp and 539bp, respectively.
MYC-2: F 5’-CGGTTCATTCTCAGACCCAGAATCC-3’; R 5’-GACATATCTCCTCCACTAGCACTCG-3’,593bp.
MYC-3: F 5’-GTGAACCGACGAACACCGGAATC-3’;
R 5’-GATTTGACCTTTCAAGGTGGGTTG-3’,386bp.
MYC-4: F 5’-GGTGTTGACTCTGGTCTTGTAGCTG-3’;
R 5’-CAACCAGAACTCTGTTACTCTCAG-3’,350bp.
CMA-A: F 5’-GTGGCGCAACACCGCCTTTCAATGC-3’;
R 5’-GTTCGACCTCGGCGATCTCCACC-3’, 488bp.
GYRA: F 5’-GAAGAGCGCGTGGTCACCATCTCC-3’; R 5’-GGAGAACAGCATGATTTCCTGCTCG-3’, 602bp.
COR-R: F 5’-GTCAGCGAAGCAAGTAGGATCGAGC-3’; R 5’-TGGAGGATGGTCAGTATCGCCTGG-3’, 446bp.
COR-S: F 5’-GGACCTATCACGCATCGAACTAGGAG-3’; R 5’-CCCTCATCACCACTCCGTTGTCG-3’,
Primers used for RT-PCR and their respective amplicon size:
ACTIN2: F 5’-AGTGGTCGTACAACCGGTATTGT -3’;
R 5’-GATGGCATGAGGAAGAGAGAAAC-3’; 93bp.
JAZ5: F 5’-CAAAGTCAAAGATGTTGCTGACC-3’;
R 5’-TCAGTAGGCTCATTGAGATCAGG-3’, 360bp.
JAZ10 (10.1,10.3 and 10.4): F 5’-AGCCTCAGATCCCGATTTCTC-3’;
R 5’-GCTGCTTCATTAGCGACCTT-3’, 166bp.
JAZ10.1: F 5’-AGCCTCAGATCCCGATTTCTC-3’;
R 5’-GATGTTG ATACTAATCTCTCCTTG -3’, 334bp.

JAZ10.3: F 5'-AGCCTCAGATCCCGATTTCTC-3';

R 5'-GGATTGTTGAAGAATCATTACCTC -3', 341bp.

JAZ10.4: F 5'-AGCCTCAGATCCCGATTTCTC-3';

R 5'-CGATGGGAAGATCGAAAGATC-3', 218bp.

MYC-2: F 5'-GAGGTAGATGGTTTTGAACGA -3';

R 5'-ACCGTTTGCTGGCTTTCTTCC-3', 150 bp.

MYC-3: F 5'-GGTAATAACTCGAATTCTAATTC-3';

R 5'-GAATGATCCGAATCATTCGCAGCTG-3', 167bp.

MYC-4: F 5'-GAAGCTCTGTTGAAAACCCTAAC-3';

R 5'-GATTCGAGTCACATGGAAGAACAG-3', 176bp.

CMA-A: F 5'- GCTGTTGGCCAATCCTCGCATTACC-3';

R 5'-GTCGAGGTAACCTAGGGCTAGATTCG -3'; 239bp.

GYRA: F 5'-TCGTCCGCTGTCAACCTGCTGC-3';

R 5'-CTTCTTCTTGTCGGCAGCCTTGATGC-3', 198bp.

COR-R: F 5'-GAAGGCATCGCCTTGTTGCAAGAGC-3';

R 5'-GGGAGGCTCTCCAGAGAGCAGC-3', 183bp.

COR-S: F 5'-CGCACGCTGGATACGGAGCTATTG-3';

R 5'-GTGTCGACGACCTCGATCTGAAGG-3',161bp.

References

Breeze E, Harrison E, McHattie S, Hughes L, Hickman R, Hill C, Kiddle S, Kim YS, Penfold CA, Jenkins D *et al*. 2011. High-resolution temporal profiling of transcripts during *Arabidopsis* leaf senescence reveals a distinct chronology of processes and regulation. *Plant Cell* **23:** 873–894.

Fernandez-Calvo P, Chini A, Fernandez-Barbero G, Chico JM, Gimenez-Ibanez S, Geerinck J, Eeckhout D, Schweizer F, Godoy M, Franco-Zorrilla JM *et al*. 2011. The Arabidopsis bHLH transcription factors MYC3 and MYC4 are targets of JAZ repressors and act additively with MYC2 in the activation of jasmonate responses. *Plant Cell* **23:** 701–715.

Gimenez-Ibanez S, Boter M, Fernandez-Barbero G, Chini A, Rathjen JP, Solano R. 2014. The bacterial effector HopX1 targets JAZ transcriptional repressors to activate jasmonate signaling and promote infection in *Arabidopsis*. *PLoS Biol* **12:** e1001792.

de Torres Zabala M, Bennett MH, Truman WH, Grant MR. 2009. Antagonism between salicylic and abscisic acid reflects early host–pathogen conflict and moulds plant defence responses. *Plant J*. **59:** 375–386.
